# Satellite glial GPR37L1 and its ligand maresin 1 regulate potassium channel signaling and pain homeostasis

**DOI:** 10.1172/JCI173537

**Published:** 2024-03-26

**Authors:** Sangsu Bang, Changyu Jiang, Jing Xu, Sharat Chandra, Aidan McGinnis, Xin Luo, Qianru He, Yize Li, Zilong Wang, Xiang Ao, Marc Parisien, Lorenna Oliveira Fernandes de Araujo, Sahel Jahangiri Esfahani, Qin Zhang, Raquel Tonello, Temugin Berta, Luda Diatchenko, Ru-Rong Ji

**Affiliations:** 1 Center for Translational Pain Medicine, Department of Anesthesiology, Duke University Medical Center, Durham, North Carolina, USA.; 2 Faculty of Dental Medicine and Oral Health Sciences, Department of Anesthesia, Faculty of Medicine and Health Science, Alan Edwards Centre for Research on Pain, McGill University, Montreal, Canada.; 3 Department of Anesthesiology, Duke University Medical Center, Durham, North Carolina, USA.; 4 Pain Research Center, Department of Anesthesiology, University of Cincinnati Medical Center, Cincinnati, Ohio, USA.; 5 Department of Neurobiology and; 6 Department of Cell Biology, Duke University Medical Center, Durham, North Carolina, USA.

**Keywords:** Neuroscience, G protein&ndash;coupled receptors, Homeostasis, Neurological disorders

## Abstract

G protein–coupled receptor 37-like 1 (GPR37L1) is an orphan GPCR with largely unknown functions. Here, we report that *Gpr37l1*/*GRP37L1* ranks among the most highly expressed GPCR transcripts in mouse and human dorsal root ganglia (DRGs) and is selectively expressed in satellite glial cells (SGCs). Peripheral neuropathy induced by streptozotoxin (STZ) and paclitaxel (PTX) led to reduced GPR37L1 expression on the plasma membrane in mouse and human DRGs. Transgenic mice with *Gpr37l1* deficiency exhibited impaired resolution of neuropathic pain symptoms following PTX- and STZ-induced pain, whereas overexpression of *Gpr37l1* in mouse DRGs reversed pain. GPR37L1 is coexpressed with potassium channels, including KCNJ10 (Kir4.1) in mouse SGCs and both KCNJ3 (Kir3.1) and KCNJ10 in human SGCs. GPR37L1 regulates the surface expression and function of the potassium channels. Notably, the proresolving lipid mediator maresin 1 (MaR1) serves as a ligand of GPR37L1 and enhances KCNJ10- or KCNJ3-mediated potassium influx in SGCs through GPR37L1. Chemotherapy suppressed KCNJ10 expression and function in SGCs, which MaR1 rescued through GPR37L1. Finally, genetic analysis revealed that the *GPR37L1-E296K* variant increased chronic pain risk by destabilizing the protein and impairing the protein’s function. Thus, GPR37L1 in SGCs offers a therapeutic target for the protection of neuropathy and chronic pain.

## Abstract 2

Satellite glial cells express GPR37L1, which protects against neuropathic pain via the regulation of potassium channel function.

## Graphical abstract

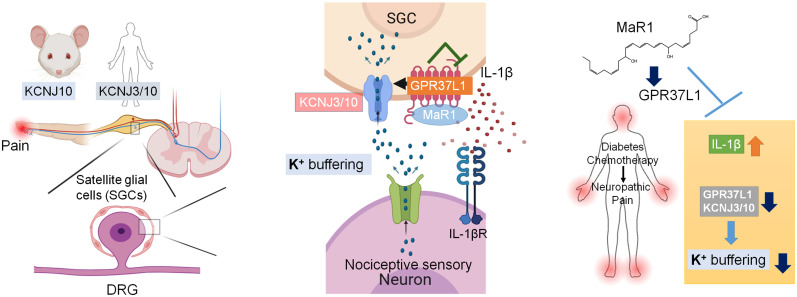

## Introduction

Satellite glial cells (SGCs) reside in the peripheral nervous system (PNS), where they wrap around neuronal cell bodies and form a complete envelope, allowing for close neuron-SGC interactions in the dorsal root ganglia (DRGs) and trigeminal ganglia (TG) (1, 2). SGCs express high levels of the inwardly rectifying K^+^ channel KCNJ10 (also known as Kir4.1) in mouse sensory ganglia, enabling them to control perineural potassium homeostasis and neuronal excitability (2, 3). Increasing evidence indicates that SGCs participate in the generation and maintenance of chronic pain (4–6). Especially, KCNJ10 is downregulated under pathological pain conditions (7), and the knockdown of KCNJ10 expression in SGCs is sufficient to induce pain hypersensitivity (3). However, it is unclear how KCNJ10 expression is regulated in SGCs and disease conditions. It is generally believed that SGCs promote pain by ATP signaling or releasing proinflammatory cytokines, such as TNF-α and IL-1β, which can drive hyperexcitability of surrounding sensory neurons (2, 8, 9).

G protein–coupled receptor 37-like 1 (GPR37L1) is an orphan GPCR. Prosaposin and prosaposin-derived peptides such as TX-14 were proposed as ligands of GPR37 and GPR37L1 and have exhibited neuroprotective and glioprotective effects, but their binding sites on GPR37L1 are elusive (10, 11). GPR37L1 was shown to have constitutive activities modulated by protease cleavage (12) and remains unliganded (13). *GPR37L1* mutations have been implicated in neurological diseases, such as seizure susceptibility (14–16). Single-cell analysis revealed that the *Gpr37l1* transcript is highly enriched in astrocytes and SGCs (17–22). However, the role of GPR37L1 in SGCs is unclear.

Specialized proresolving mediators (SPMs), such as resolvins, protectins, and maresins, are biosynthesized from omega-3 unsaturated fatty acids (e.g., docosahexaenoic acid [DHA]) and exhibit potent proresolution, antiinflammation, and analgesic actions in various animal models via GPCR activation (23–27). We previously identified neuroprotectin D1 (NPD1) as a ligand for GPR37 (28). In this study, we identified the proresolution lipid mediator maresin 1 (MaR1) as a ligand of GPR37L1. We found that MaR1 interacts with and binds GPR37L1. MaR1 potently attenuated neuropathic pain and further increased KCNJ10-mediated K^+^ currents in SGCs in a GPR37L1-dependent manner. We also revealed a distinct expression of KCNJ3 (Kir3.1/GIRK1) in humans but not in mouse SGCs and demonstrated that MaR1 increased the KCNJ3 activity in human SGCs. Finally, we identified what we believe are previously unrecognized *GPR37L1* variants that are associated with chronic pain in humans.

## Results

### Gpr37l1 and GPR37L1 are highly expressed in SGCs of mouse and human DRGs.

**Figure 1:**
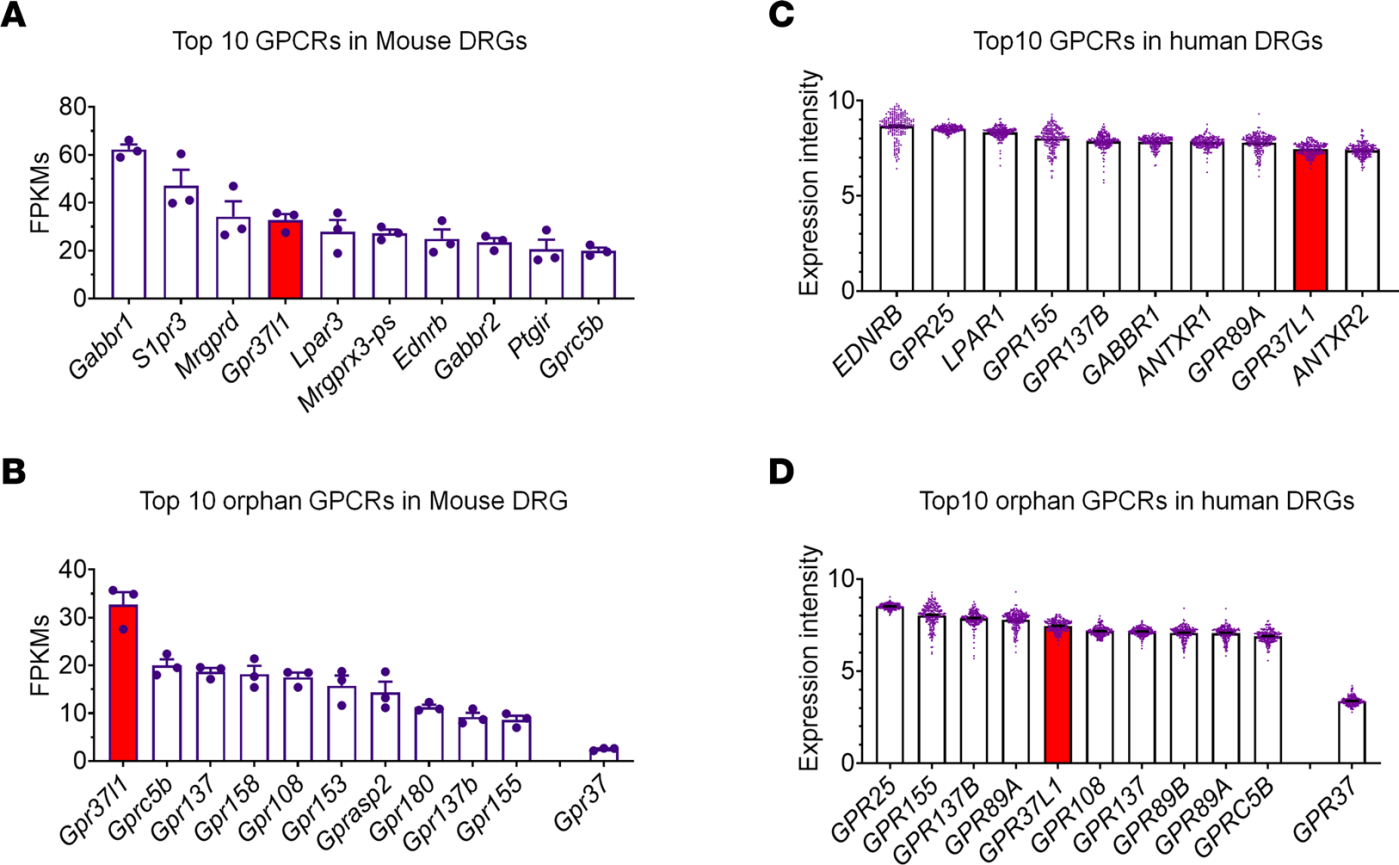
*Gpr37l1* and *GPR37L1* transcripts are highly expressed in mouse and human DRGs. (**A** and **B**) Highly expressed GPCR transcripts in mouse DRGs. RNA-Seq shows mRNA expression levels (FPKM) of the top 10 GPCR transcripts (**A**, *n* = 3) and the top 10 orphan GPCR transcripts (**B**, *n* = 3). *Gpr37l1* expression is highlighted in red bars. Note that the *Gpr37* expression is much lower than *Gpr37l1*. (**C** and **D**) Highly expressed GPCR transcripts in human DRGs. Normalized microarray shows mRNA expression levels (intensity) of the top 10 GPCR transcripts (**C**, *n* = 214) and the top 10 orphan GPCR transcripts (**D**, *n* = 214). *GRP37L1* expression is highlighted in red bars. *GPR37* expression is included for comparison. Data are represented as means ± SEM.

We conducted RNA-Seq from DRGs of noninjured naive mice and detected a total of 321 GPCRs and 92 orphan GPCRs with a cutoff value of more than 1 (Supplemental Table 1A; supplemental material available online with this article; https://doi.org/10.1172/JCI173537DS1). Figure 1, A and B, shows the top 10 expressed GPCR transcripts in both categories. We found that *Gpr37l1* is among the 10 most highly expressed GPCR mRNAs (Figure 1A; *n* = 3). *Gpr37l1* is also the most expressed orphan GPCR mRNA in mouse DRGs (Figure 1B and Supplemental Table 1B*, n* = 3). We next examined GPCR mRNA expression levels in human DRGs using a microarray database with a total of 332 GPCR and 73 orphan GPCR transcripts (Supplemental Table 2A; *n* = 214). Figure 1, C and D, shows the 10 most highly expressed GPCR transcripts in both categories. We found that *GPR37L1* is among the top 10 GPCR mRNAs (Figure 1C and Supplemental Table 2A) and the top 5 orphan GPCR mRNAs in human DRGs (Figure 1D and Supplemental Table 2B). Therefore, *Gpr37l1* and *GPR37L1* mRNAs are highly expressed by both mouse and human DRGs. See the NCBI’s Gene Expression Omnibus database for mouse and human DRG-sequencing data, respectively (GEO GSE260669 and GSE78150).

**Figure 2:**
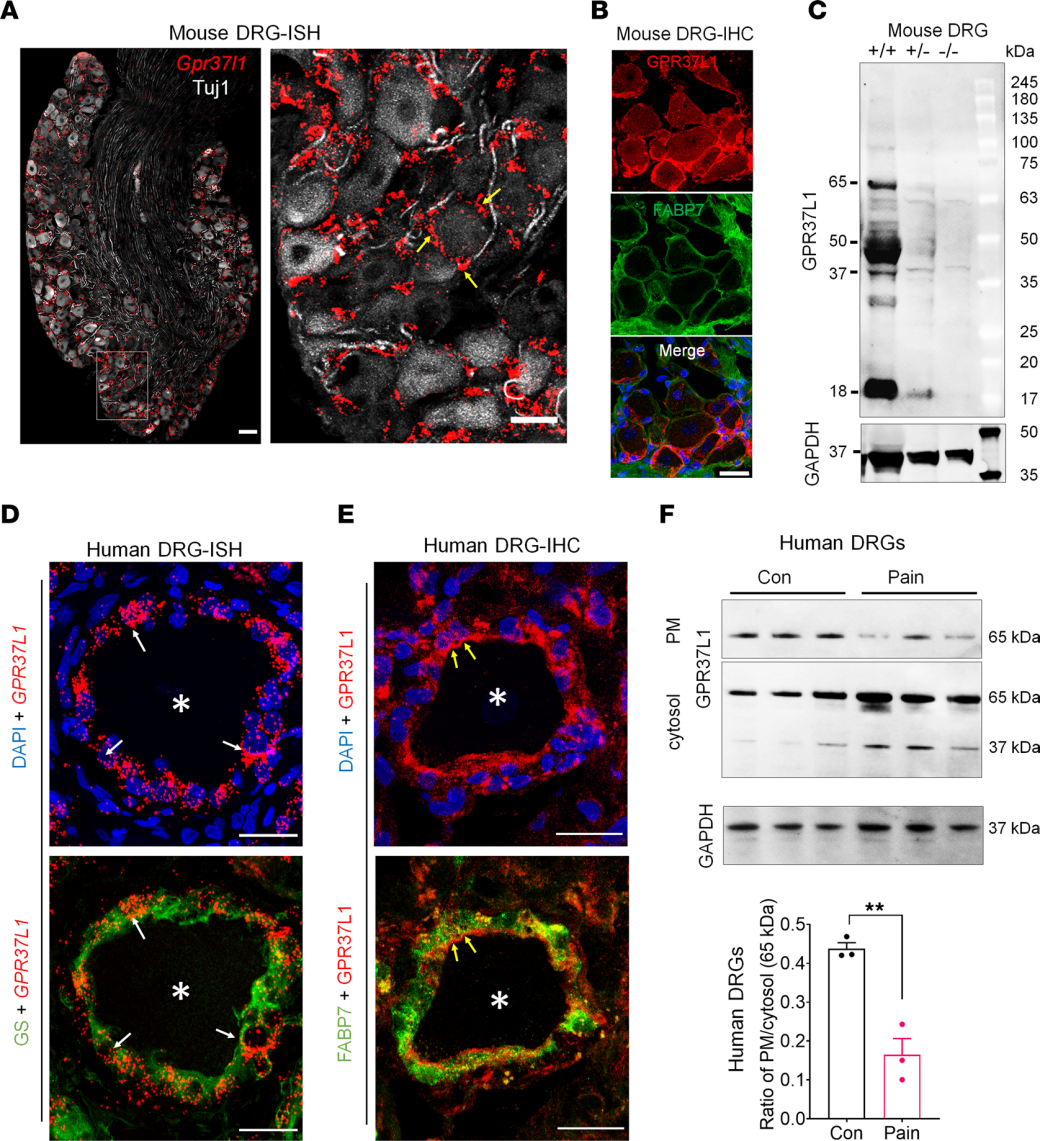
Mouse and human SGCs express *Gpr37l1*/*GPR37L1* mRNA and GPR37L1 protein. (**A**) Double staining of RNAscope ISH (for *Gpr37l1*) and IHC (for Tuj1) show nonoverlapping expression of *Gpr37l1* mRNA (red) and Tuj1 (white) in mouse DRGs. Right, enlarged image from the box in the left panel. Yellow arrows indicate *Gpr37l1*^+^ cells surrounding DRG neurons. Scale bars: 25 μm. (**B**) Double IHC staining shows colocalization of GPR37L1 (red) with FABP7 (white). Scale bar: 25 μm. (**C**) Western blot showing GPR37L1 expression in DRGs of *Gpr37l1^+/+^, Gpr37l1^+/–^, and Gpr37l1^–/–^* mice. GAPDH was included as a loading control from the same gel. (**D**) Double staining of ISH (*GPR37L1,* red) and IHC (GS green) shows colocalization of *GPR37L1* mRNA and GS in human SGCs. (**E**) Double staining of IHC for GPR37L1 (red) and FABP7 (green) shows heavy colocalization of GPR37L1 and FABP7 in human SGCs. Note that GPR37L1 is enriched on the inner side of SGCs in close contact with neurons. Asterisks indicate neurons, and arrows indicate SGCs surrounding the neurons. Scale bars: 25 μm. (**F**) Top, Western blots showing PM and intracellular cytosol (IC) fractions of GPR37L1 and GAPDH loading control (from the same gel) in human DRGs of neuropathic pain patients and controls (Con) (*n* = 3). Bottom, the ratio of PM/IC GPR37L1 expression in human DRGs of control and neuropathic pain patients. Data are represented as means ± SEM and analyzed by *t* test. **P < 0.05, unpaired Student’s *t* test. *n* = 3.

Single-cell RNA-Seq has revealed selective expression of *Gpr37l1* mRNA in mouse SGCs of mouse DRG (17, 19–21, 29, 30) (Supplemental Figure 1A). Next, we investigated *Gpr37l1* mRNA and GPR37L1 protein expression in mouse DRGs using RNAscope ISH, IHC, Western blotting, and flow cytometry in both WT and *Gpr37l1* mutant mice (Supplemental Figure 2, A–D). ISH analysis revealed *Gpr37l1* expression in SGCs but not neurons of mouse DRG (Figure 2A). Additionally, SGCs of TG and nodose ganglia expressed *Gpr37l1* (Supplemental Figure 1, B and C). *Gpr37l1* expression was absent in *Gpr37l1^–/–^* (KO) mice, validating the specificity of the RNAscope probe (Supplemental Figure 1D). Notably, *Gpr37*, a close family member of *Gpr37l1*, is expressed by neurons but not by SGCs (Supplemental Figure 1E) in mouse DRGs. Double staining via IHC showed colocalization of GPR37L1 with FABP7, a cellular marker for SGCs (19). Notably, GPR37L1 staining formed a ring surrounding DRG neurons (Figure 2B). Western blot analysis detected several forms of GPR37L1 in mouse DRGs, a full-length GPR37L1 at approximately 65 kDa (glycosylated form), and an approximately 50 kDa band (nonglycosylated form) as well as truncated/cleaved forms of GPR37L1 at 37 and 18 kDa (Figure 2C). These bands were substantially reduced in the heterozygote (*Gpr37L1^+/–^*) mice and completely abolished in homozygote (*Gpr37l1^–/–^*) mice (Figure 2C). Flow cytometry analysis showed colocalization of GPR37L1 with approximately 80% of GLAST^+^ cells in WT DRGs (Supplemental Figure 2, A and B). GPR37L1 expression was abolished in *Gpr37l1^–/–^* mice (*P* < 0.0001, Supplemental Figure 2B) and also significantly decreased in mice treated with intraganglionic (i.g.) injection of *Gpr37l1* siRNA (*P* < 0.001, Supplemental Figure 2C).

We also conducted ISH, IHC, and Western blotting to examine GPR37L1 expression in human DRG tissues. Double staining of ISH and IHC revealed that the *GPR37L1* transcript is specifically expressed in human SGCs that coexpress the SGC marker glutamine synthetase (GS) (2) (Figure 2D). Furthermore, double-IHC staining showed heavy colocalization of GPR37L1 with FABP7. Intriguingly, GPR37L1 expression on SGCs appears to be polarized: it is highly visible on the inner side of the SGC that is in close contact with neurons (Figure 2E). This unique subcellular expression provided an anatomical substrate for GPR37L1 to mediate neuron-glial interaction.

To further the translational relevance of this study, we analyzed human DRG samples from patients with diabetic peripheral neuropathy (DPN). Western blot analysis revealed a downregulation of plasma membrane GPR37L1 (GPR37L1-PM) but an upregulation of intracellular cytosol GPR37L1 (GPR37L1-IC), leading to a significant change in PM/IC ratio (*P* < 0.05, Figure 2F). This result suggests that GPR37L1 is regulated in painful disease conditions such as diabetic neuropathy.

### GPR37L1 is protective against PTX- and STZ-induced pain model.

**Figure 3:**
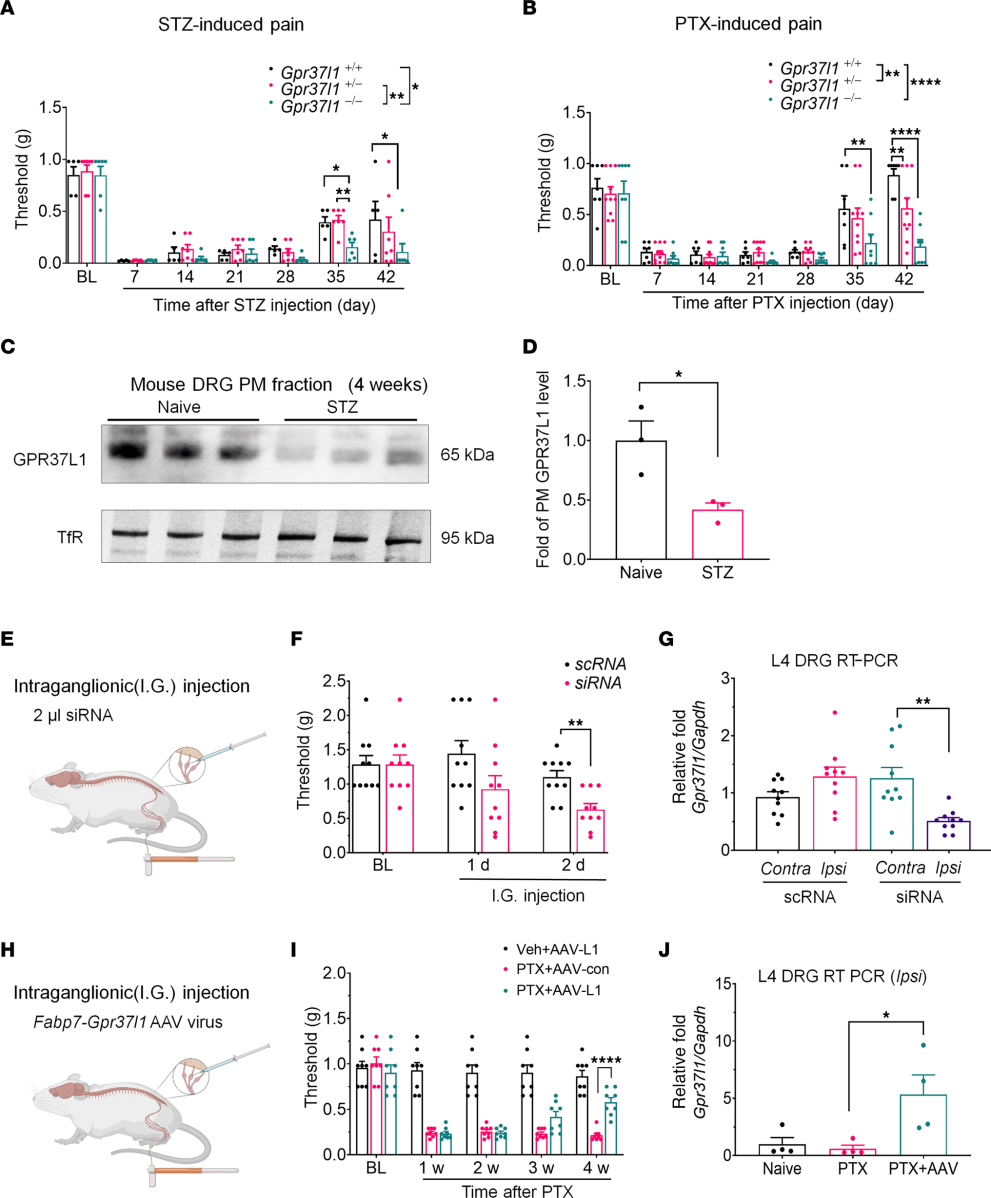
GPR37L1 is dysregulated in pain and protects against neuropathic pain. (**A** and **B**) Neuropathic pain (mechanical allodynia) induced by STZ (75 mg/kg) and PTX (6 mg/kg) in WT and *Gpr37l1* mutant mice. (**A**) Time course of STZ (75mg/kg)-induced mechanical allodynia in *Gpr37l1^+/+^* mice (*n* = 5)*, Gpr37l1^+/–^* mice (*n* = 7), and *Gpr37l1^–/–^* mice (*n* = 6). (**B**) Time course of PTX (6 mg/kg)-induced mechanical allodynia in *Gpr37l1^+/+^* mice (*n* = 7)*, Gpr37l1^+/–^* mice (*n* = 10), and *Gpr37l1^–/–^* mice (*n* = 8). (**C** and **D**) GPR37L1 expression in PM fraction of DRG tissues of control mice and mice with STZ treatment (*n* = 3). Transferrin (TfR) was used as a loading control from a parallel gel. (**D**) Quantification of GPR37L1. (**E**–**G**) Unilateral i.g. microinjection of *Gpr37l1*-targeting siRNA reduces *Gpr37l1* expression and induces persistent mechanical allodynia in naive animals. (**E**) Schematic of i.g. microinjection of siRNA or scRNA in the L4 and L5 DRGs, followed by von Frey testing and tissue collection for quantitative reverse-transcription PCR (RT-PCR) analysis. (**F**) Mechanical allodynia is induced by siRNA (*n* = 10), not scRNA (*n* = 10). (**G**) RT-PCR analyses of *Gpr37l1* in DRGs (*n* = 10 mice). (**H**–**J**) Unilateral i.g. microinjection of *Fabp7*-*Gpr37l1* (AAV-L1) or Fabp7-mock AAV virus (AAV-Con) rescued *Gpr37l1* expression and reduced persistent mechanical allodynia in CIPN mice (6 mg/kg PTX). (**H**) Schematic of i.g. microinjection of AAV virus in the L4 and L5 DRGs, given 1 week after PTX, followed by von Frey testing and tissue collection for RT-qPCR analysis. (**I**) PTX-mediated mechanical allodynia is reduced by AAV-L1 application (*n* = 8) but not AAV-Con (*n* = 8). (**J**) RT-PCR analyses showing expression of *Gpr37l1* (*n* = 4 mice). Data are represented as mean ± SEM and statistically analyzed by 2-way ANOVA with Tukey’s post hoc test (**A**, **B**, and **I**) or Bonferroni’s post hoc test (**F**), 1-way ANOVA with Tukey’s post hoc test (**G** and **J**), and 2-tailed *t* test (**D**). *P < 0.05; **P < 0.01; ****P < 0.0001.

To evaluate the contribution of GPR37L1 to neuropathic pain, we generated 2 animal models by systemic injection of streptozotoxin (STZ), a diabetes-inducing toxin, and paclitaxel (PTX), a chemotherapy drug. We tested the time course of STZ- and PTX-induced mechanical allodynia, a cardinal feature of neuropathic pain in these mouse models in 3 genotypes: *Gpr37l1^+/+^*, *Gpr37l1^+/–^*, and *Gpr37l1^–/–^*. Notably, the baseline pain sensitivity, including mechanical, heat, and cold sensitivity, did not differ among the 3 genotypes (Supplemental Figure 3, A–C). A low dose of STZ (75 mg/kg) evoked rapid mechanical allodynia in 7 days, and this mechanical pain resolved on day 35 (Figure 3A). Interestingly, *Gpr37l1^–/–^* mice failed to resolve on day 35 and day 42 compared with *Gpr37l1^+/+^* mice (*P* < 0.05 on day 35 and day 42, Figure 3A). A single injection of PTX (6 mg/kg) also elicited profound mechanical allodynia in 7 days, and this allodynia resolved on day 35 (Figure 3B). Notably, *Gpr37l1^–/–^* mice showed no sign of resolution on day 35 and day 42 compared with *Gpr37l1^+/+^* mice (*P* < 0.01 on day 35, *P* < 0.0001 on day 42; Figure 3B). Collectively, the results indicate a protective role of GPR37L1 against STZ- and PTX-induced pain. Consistent with this notion, the STZ-induced pain model was associated with a significant reduction of surface expression of GPR37L1 in the PM of mouse DRGs collected 28 days after the STZ injection (*P* < 0.05, Figure 3, C and D).

To determine whether downregulation of GPR37L1 is sufficient to produce pain, we conducted intraganglionic microinjection of *Gpr37l1*-targeting siRNA (siRNA) or control scramble RNA (scRNA) to the L4 and L5 DRGs (4 µg in 2 µl per injection, Figure 3E). We found that this siRNA treatment was sufficient to induce mechanical allodynia within 2 days (Figure 3F and Supplemental Figure 3D), with mild effects on thermal sensitivity (Supplemental Figure 3, E and F). Compared with scRNA, siRNA-treated mice had an approximately 60% reduction in *Gpr37l1* mRNA levels in the L4–L5 DRGs 2 days after the injection (*P* < 0.001, versus scRNA, Figure 3G). A similar reduction in GPR37L1 expression was validated by flow cytometry (Supplemental Figure 2C). This finding suggests that a partial loss of GPR37L1 in L4–L5 DRGs is sufficient to drive pain.

Next, we tested to determine whether an upregulation of GPR37L1 in DRG SGCs is sufficient to rescue neuropathic pain. To this end, we conducted intraganglionic microinjection of SGC-targeting *Gpr37l1-*AAV9 virus (with *Fabp7* promoter) and control AAV9 virus (1 × 10^12^ CFU in 1 µl) to the L4 and L5 DRGs, given 1 week after PTX injection (Figure 3H). The *Gpr37l1*-AAV9 treatment resulted in a delayed reversal of mechanical allodynia 4 weeks after the PTX injection.

The Gpr37l1-AAV9 treatment reversed mechanical allodynia caused by PTX application at 4 weeks. (*P* < 0.0001 versus control AAV9, Figure 3I). As expected, *Gpr37l1*-AAV9 treatment also resulted in a substantial increase in *Gpr37l1* mRNA levels in L4 DRGs at 4 weeks after PTX injection (*P* < 0.05, Figure 3J). Together, both loss-of-function and gain-of-function experiments support the beneficial role of GPR37L1 in pain protection.

### MaR1 serves as a GPR37L1 ligand.

**Figure 4:**
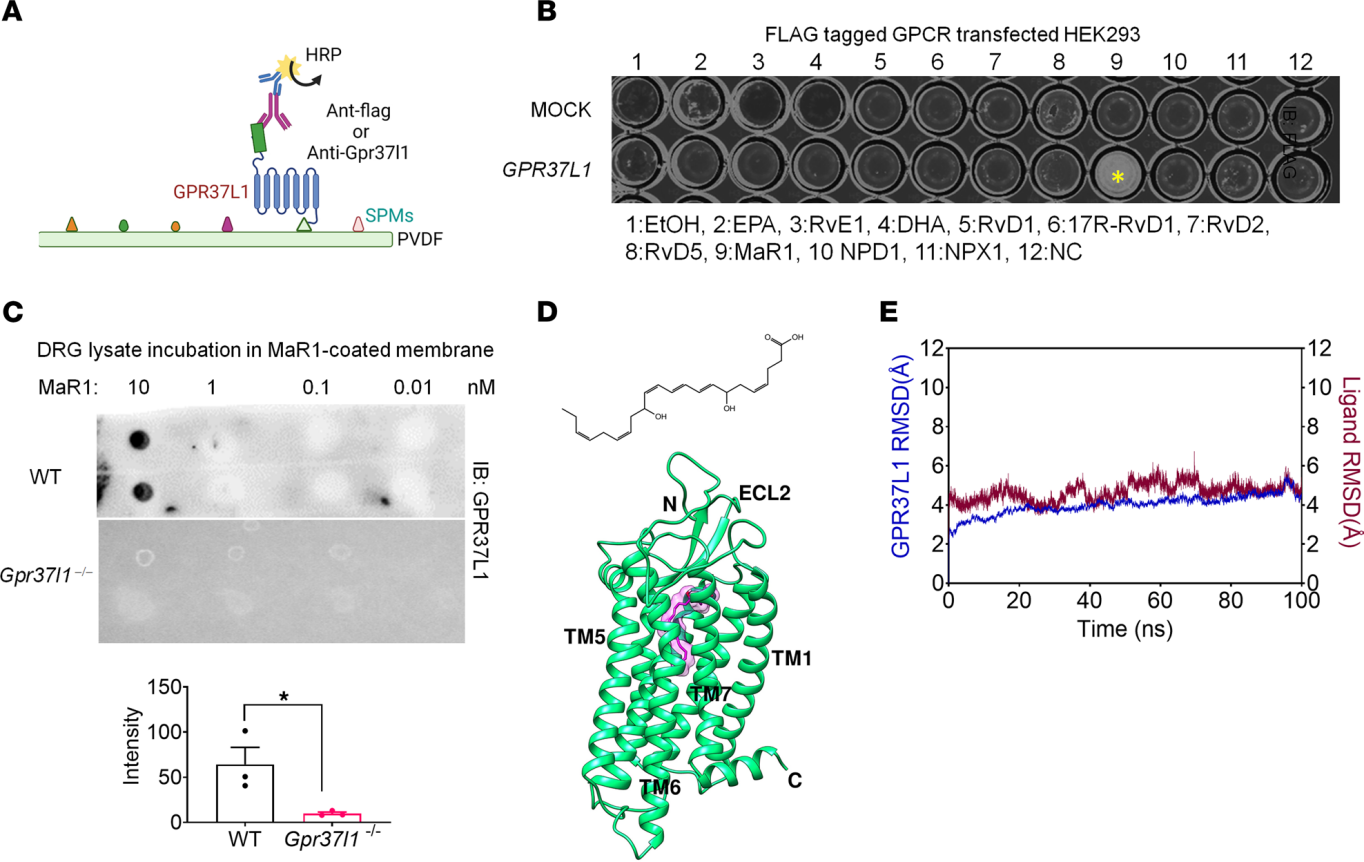
MaR1 binds GPR37L1. (**A** and **B**) Lipid overlay assay shows GPR37L1 binding to MaR1. (**A**) Schematic for detecting GPR37L1-binding lipid mediators. hGPR37L1 is FLAG tagged and expressed in HEK293 cells after *hGPR37L1* cDNA transfection. (**B**) Specific binding of hGPR37L1 to MaR1. The plate was coated with 10 different lipid mediators (1 µg/ml, nos. 2–11) as well as vehicle control (0.1% ethanol, no. 1) and no coating control (NC, no. 12) and then incubated with cell lysates of *hGPR37L1*- or mock-transfected cells. Yellow asterisk indicates a positive response. (**C**) Upper panel: representative blot of MaR1-coated PVDF membrane. Lower panel: quantification of dot intensity in DRG lysates from WT and *Gpr37l1^–/–^* mice. *n* = 3 repeats. (**D** and **E**) Overall structure of hGPR37L1 (green) in complex with MaR1 (magenta). (**E**) 100 ns RMSD graph was obtained with a 1000 ns simulation of the GPR37L1-MaR1 complex (red) or GPR37L1(blue). Data are expressed as means ± SEM and were analyzed by unpaired *t* test (**C**) *P < 0.05.

SPMs, such as resolvins, protectins, and maresins, are biosynthesized from omega-3 unsaturated fatty acids, such as DHA, and demonstrate potent analgesic actions in various animal models via activation of specific GPCRs (23–27). We previously identified NPD1 as a ligand for GPR37 (28). Since GPR37L1 is a close family member of GPR37, we speculated that SPMs may also activate GPR37L1. We tested different families of SPMs, including D-series resolvins (RvD1, RvD2, and RvD5), E-series resolvin (RvE1), NPD1, and MaR1 as well as the SPM precursors DHA and eicosapentaenoic acid (EPA). This was accomplished by using a lipid overlay assay to detect binding partners of GPR37L1, which we established by transfecting HEK cells with *GPR37L1* containing a FLAG tag (Figure 4A). Among all the lipid mediators we tested, only MaR1 showed a specific binding signal to GPR37L1; no signal was detected following mock transfection (Figure 4B). To confirm whether native GPR37L1 expressed by mouse DRGs would also interact with MaR1, we performed a lipid overlay assay using DRG lysates from WT or *Gpr37l1^–/–^* mice (Figure 4C). We coated PVDF membranes with MaR1 (0.01, 0.1, 1, and 10 nM) and observed specific binding of 10 nM MaR1 in DRG samples from WT but not from *Gpr37l1^–/–^* mice (*P* < 0.05, WT versus KO, Figure 4C). Furthermore, we performed a lipid-coated bead pull-down assay to determine whether MaR1 would interact with the GPR37L1 protein. We coated beads with MaR1, RvD1, RvD2, NPD1, and DHA and found strong GPR37L1 binding to MaR1 (Supplemental Figure 4, A–C). These results suggested a direct binding of MaR1 to GPR37L1.

Using GPR37L1 homology modeling, we generated computational predictions to examine the potential interaction between MaR1 and GPR37L1. The molecular structures for GPR37L1 and GPR37 have not been solved, but these 2 GPCRs have high homologies with human endothelial receptor type B (human EDNRB, Supplemental Figure 5A) (31). Thus, we conducted EDNRB transmembrane helix sequence alignment with human GPR37L1 and utilized the crystal structure of human EDNRB (PDB: 6IGK) as a template for GPR37L1 structural modeling (Figure 4D). We investigated possible interactions of MaR1 and NPD1 (control) with GPR37L1 by assessing the strength of hydrogen bonds between them using molecular docking and molecular dynamic simulation (MDS) (Figure 4E and Supplemental Figure 5, B and C). In the MDS of the GPR37L1-MaR1 complex cluster 1, the RMSD value of the protein backbone was stabilized at 4Å and the RMSD of MaR1 was between 4 and 6Å (Supplemental Figure 5C). Further 100 ns simulation of the GPR37L1-MaR1 cluster-1 ensemble structure also demonstrated stable interactions, with RMSD values between 4 and 6Å (Figure 4D). In contrast, NPD1 does not show stable MDS activity for GPR37L1 (Supplemental Figure 5, B and D), even though NPD1 can bind GPR37 (31).

### MaR1 inhibits STZ- and PTX-induced pain via SGC and GPR37L1 signaling.

**Figure 5:**
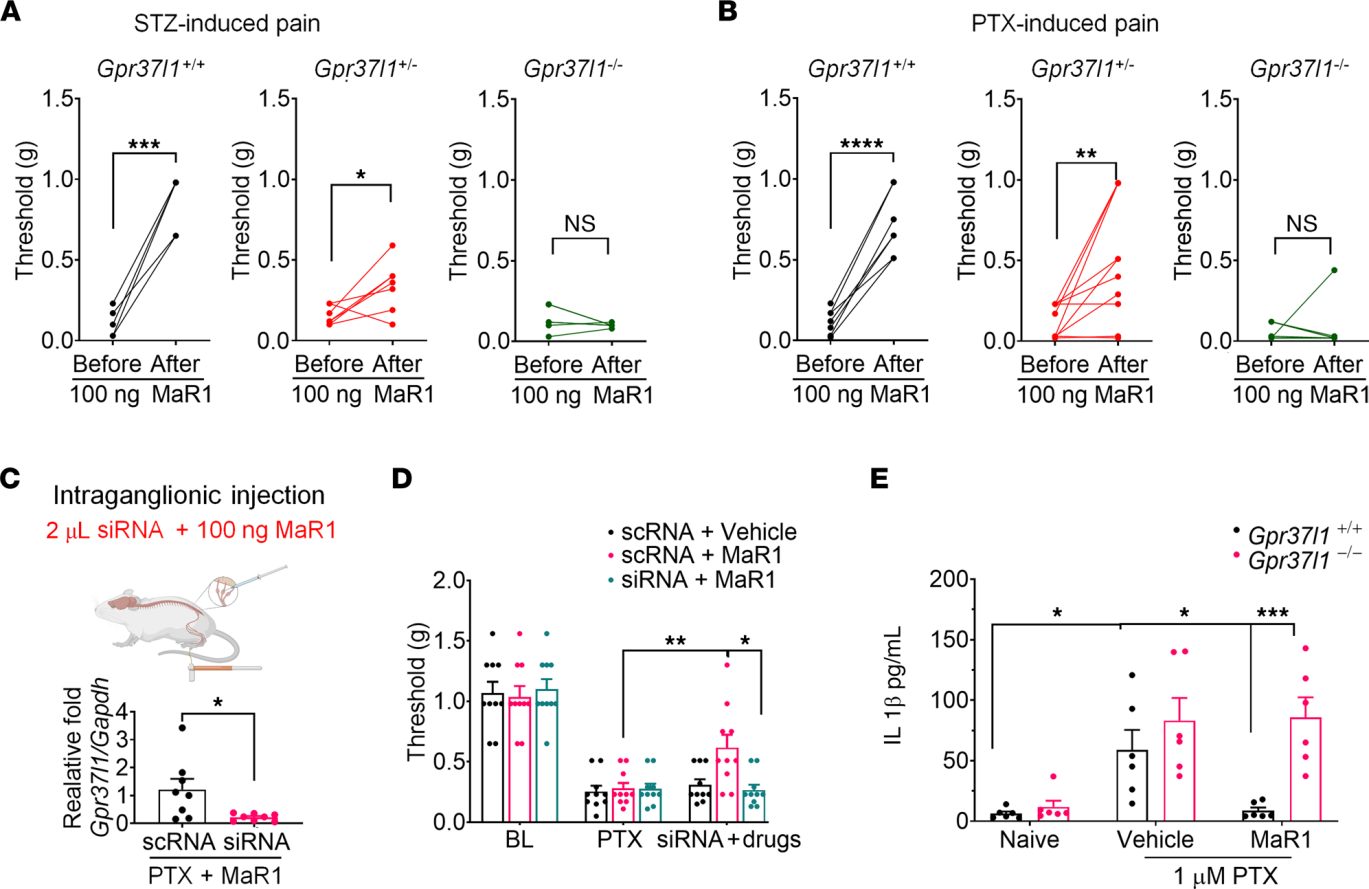
Injection i.t. or i.g. of MaR1 reduces neuropathic pain via GPR37L1 expressed on SGCs. (**A** and **B**) MaR1 injected i.t. (100 ng) reduces mechanical allodynia in *Gpr37l1^+/+^* and *Gpr37l1^+/–^* mice induced by 75 mg/kg of STZ and 6 mg/kg of PTX. (**A**) Paw withdrawal thresholds were assessed in *Gpr37l1^+/+^* mice (left, *n* = 7), *Gpr37l1^+/–^* mice (middle, *n* = 10), and *Gpr37l1^–/–^* mice (*n* = 8) after i.t. MaR1 injection in the STZ model. (**B**) Paw withdrawal thresholds in *Gpr37l1^+/+^* mice (left, *n* = 5), *Gpr37l1^+/–^* mice (middle, *n* = 7), and *Gpr37l1^–/–^* mice (*n* = 6) after i.t. MaR1 injection in the PTX model. Behavior was assessed 1 hour after MaR1 injection on post-PTX and post-STZ day 3. (**C**) Upper panel, schematic of i.g. injection. Lower panel, knockdown of *Gpr37l1* expression in the L4 DRGs after siRNA treatment compared with scRNA treatment (*n* = 8). (**D**) Injection of MaR1 (i.g., 10 ng, 2 µl) reduces PTX-induced mechanical allodynia in control animals treated with scRNA, but not in animals treated with *Gpr37l1* siRNA (*n* = 10). (**E**) MaR1 inhibits PTX-induced IL-1β release in SGC-neuron cocultures via GPR37L1. IL-1β release in cocultures from DRG of WT mice (*n* = 6) and *Gpr37l1^–/–^* mice (*n* = 6) was analyzed by ELISA. The cultures were stimulated with 1 μM PTX for 24 hours in the absence or presence of MaR1 (100 nM). Data are expressed as means ± SEM and were statistically analyzed by paired *t* test (**A** and **B**), 2-way ANOVA with Bonferroni’s post hoc test (**D** and **E**), Tukey’s post hoc test (**D** and **E**), or unpaired *t* test (**C**). *P < 0.05, **P < 0.01, ***P < 0.001, ****P < 0.0001.

MaR1 was shown to inhibit inflammatory pain in acute and chronic pain models (24, 26). We also tested the analgesic actions of MaR1 on STZ- and PTX-induced neuropathic pain in *Gpr37l1^+/+^*, *Gpr37l1^+/–^*, and *Gpr37l1^–/–^* mice. First, we administered MaR1 via an intrathecal (i.t.) route that can target cells in DRGs and the spinal cord. We observed that, in *Gpr37l1^+/+^* (WT) mice, MaR1 significantly reversed STZ-induced mechanical allodynia in all the mice (Figure 5A, *P* < 0.001). MaR1 also significantly reversed the mechanical allodynia in *Gpr37l1^+/–^* mice (Figure 5A, *P* < 0.05). However, MaR1 had no significant analgesic effects in *Gpr37l1^–/–^* mice (Figure 5A). Furthermore, we observed similar analgesic effects of MaR1 in the chemotherapy model (Figure 5B). MaR1 significantly reversed PTX-induced mechanical allodynia in WT mice (Figure 5B, *P* < 0.0001). MaR1 also significantly reversed the mechanical allodynia in *Gpr37l1^+/–^* mice (Figure 5B, *P* < 0.01), but had no significant analgesic effects in *Gpr37l1^–/–^* mice (Figure 5B).

An i.t. injection of MaR1 could reduce pain at both DRG and spinal cord levels. To determine a direct effect of MaR1 on DRG cells, we conducted i.g. injection of MaR1 and *Gpr37l1* siRNA (Figure 5C). As expected, i.g. treatment of *Gpr37l1* siRNA resulted in a significant reduction of *Gpr37l1* mRNA expression (*P* < 0.05, vs. scRNA, Figure 5C). Notably, i.g. injection of MaR1 significantly reduced mechanical pain in chemotherapy-treated mice treated with control siRNA (scRNA, *P* < 0.01), but this analgesic effect was compromised in mice treated with *Gpr37l1*-siRNA (*P* < 0.05, Figure 5D). Collectively, these results indicate that MaR1 may relieve pain through GPR37L1 signaling in DRG.

SGCs have been shown to promote pain by releasing proinflammatory cytokines, such as IL-1β (8, 32). To examine the SGC-mediated neuro-glial interactions in pathological conditions, we prepared neuron-glia mixed cultures from the DRGs of both WT and *Gpr37l1^–/–^* mice and stimulated these cocultures with 1 μM PTX for 24 hours. ELISA showed that PTX induced a marked increase in IL-1β levels in the culture medium and that this increase was blocked by 100 nM MaR1 in cultures from WT but not *Gpr37l1^–/–^* mice (Figure 5E). Thus, MaR1 may partially alleviate neuropathic pain by suppressing IL-1β release via GPR37L1-mediated intracellular signaling in SGCs.

### MaR1 regulates KCNJ10 expression and K^+^ channel function in SGCs.

**Figure 6:**
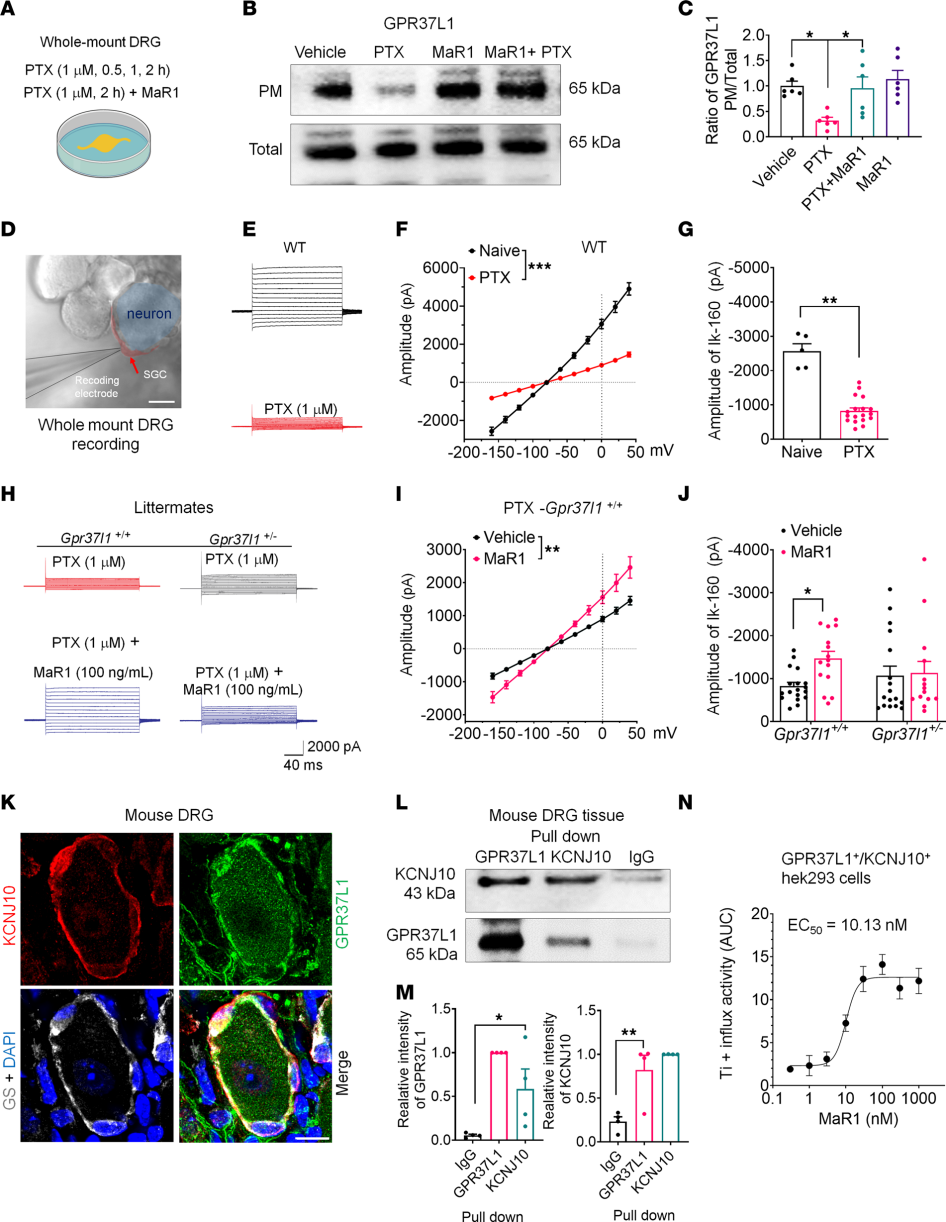
MaR1 increases surface expression of GPR37L1 and KCNJ10/Kir4.1 and regulates K^+^ currents in SGCs via GPR37L1. (**A**–**C**) Western blot analysis of surface GPR37L1 in whole-mount DRG. (**A**) Schematic of whole-mount DRG treatment with PTX or MaR1, followed by PM preparation and Western blot. (**B**) Western blot showing effects of PTX (1 μM) and MaR1 (100 ng/ml, 2 hours) on PM and total fraction of GPR37L1. (**C**) Quantification of GRP37L1 expression (*n* = 6 preps). (**D**–**J**) Patch-clamp recordings of K^+^ currents in SGCs in whole-mount DRG. (**D**) Micrograph showing patch-clamp recording in SGC (red arrow). Scale bar: 10 μm. (**E**) Representative trace for total K^+^ currents in SGCs. (**F**) Average current-voltage (I/V) curves in SGCs treated with vehicle (*n* = 5 cells) and PTX (1 μM, *n* = 18 cells). (**G**) Quantification of amplitude of K^+^ currents (I_k-160_). (**H**) Representative traces for total K^+^ currents in SGCs of *Gpr37l1^+/+^* or *Gpr37l1^+/–^* DRGs treated with PTX (1 μM, 1 hour) or PTX plus MaR1 (100 ng/ml, 1 hour) or in *Gpr37l1^+/–^* DRGs. (**I**) Average I/V curves in WT SGCs after treatment with PTX (*n* = 18 cells) or PTX plus MaR1 (*n* = 15 cells). (**J**) Amplitude of Κ^+^ currents (I_k-160_) for **H** and **I**. *Gpr37l1^+/–^* DRG preps: *n* = 17 for PTX; *n* = 14 for PTX plus MaR1. (**K**) Triple IHC staining colocalization of GPR37L1 (green), GS (white), and KCNJ10 (red) in mouse DRG SGCs. Scale bar: 25 μm. (**L**) Protein pull-down and Co-IP show GPR37L1 and KCNJ10 interaction in mouse DRGs from parallel gels. (**M**) Quantification of pull-down results in **L** for GPR37L1 and KCNJ10. *n* = 5 mice. (**N**) Dose-response curve of Ti^+^ influx based on AUC using Richard’s 5-parameter methods (10 minutes, *n* = 6). Data are expressed as mean ± SEM and were analyzed by 2-way ANOVA with Bonferroni’s post hoc test (**F**, **I**, and **J**), 1-way ANOVA with Tukey’s post hoc test (**C** and **M**), or 2-tailed *t* test (**G**). *P < 0.05; **P < 0.01; ***P < 0.001.

KCNJ10 is the predominant potassium channel in mouse SGCs (3) (Supplemental Figure 1A) and is responsible for the generation of K^+^ currents in SGCs (33). Dysregulation of KCNJ10 in SGC has been implicated in the pathogenesis of pain (2). To define the direct effect of chemotherapy on the DRG, we employed an ex vivo whole-mount DRG preparation for both biochemical and electrophysiological analyses (Figure 6A) (34). We found that PTX treatment (1 μM) caused a rapid change in PM expression (Figure 6B and Supplemental Figure 6, A and B) with significant downregulation at 2 hours (*P* < 0.05, Figure 6, B and C, and Supplemental Figure 6, A and B). Notably, 100 ng/ml MaR1 treatment was able to dramatically reverse this downregulation (*P* < 0.05, Figure 6, B and C).

To investigate K^+^ signaling in SGCs, we conducted whole-cell patch-clamp recordings of K^+^ currents in SGCs (Figure 6, D–J). We visualized SGCs with differential interference contrast (DIC) microscopy (Figure 6D). Under normal conditions, SGCs exhibited large K^+^ currents (>2 nA) at the holding potential of –160 mV (*I*_k-160_, Figure 6E and Supplemental Figure 7, A and B). PTX treatment also caused a significant downregulation of the surface expression of KCNJ10 at 2 hours (*P* < 0.001, Supplemental Figure 6, A and B). Also, PTX (100 ng, i.g. injection) induced acute pain response prevented by 100 ng MaR1 application *(*Supplemental Figure 6, C–F).

Strikingly, PTX (1 μM) induced a rapid reduction in K^+^ currents within 20 minutes (Figure 6, E–G, and Supplemental Figure 7, B–D). PTX evoked a dose-dependent effect: a significant reduction of *I*_k-160_ was observed at concentrations as low as 0.1 μM PTX (*P* < 0.05, Supplemental Figure 7E). As previously reported (35), the total K^+^ currents were almost completely blocked by the Kir channel blocker barium (100 μM); the amplitude of the Kir-mediated *I*_k-160_ current was comparable with that of the total K^+^ currents (Supplemental Figure 7, F and G). Coapplication of MaR1 (100 ng/ml) with PTX significantly reversed the K^+^ current deficit (*P* < 0.01, Figure 6, H and I). Importantly, MaR1’s enhancement of K^+^ currents was abolished in SGCs of *Gpr37l1* mutant mice (Figure 6, H and J, and Supplemental Figure 7H).

To investigate how GPR37L1 regulates KCNJ10-mediated K^+^ channel function, we performed (a) IHC to confirm GPR37L1/KCNJ10 coexpression in mouse DRG sections (Figure 6K), (b) GPR37L1/KCNJ10 Co-IP in mouse DRG tissues (Figure 6, L and M), and (c) Ti^+^ influx assay for assessing intracellular K^+^ levels in HEK293 cells transiently expressing GPR37L1 and KCNJ10 (Figure 6N and Supplemental Figure 7I). Triple-IHC staining revealed that KCNJ10 is highly coexpressed with GPR37L1 in GS^+^ SGCs (Figure 6K). Co-IP analysis revealed that GRP37L1 or KCNJ10 antibodies could pull down KCNJ10 or GPR37L1, respectively, in mouse DRG lysates (Figure 6, L and M). Ti^+^ influx assay showed a dose-dependent increase in Ti^+^ influx in HEK293 cells incubated with 0.1–1 μM (1 hour) of MaR1, with EC_50_ = 7.2 nM (Figure 6N). MaR1-induced Ti^+^ influx peaked in 10 minutes (Supplemental Figure 7J). Collectively, these results demonstrate that MaR1 regulates KCNJ10-mediated K^+^ influx through GPR37L1 signaling.

### MaR1 regulates KCNJ3 expression and K^+^ channel function in human SGCs.

**Figure 7:**
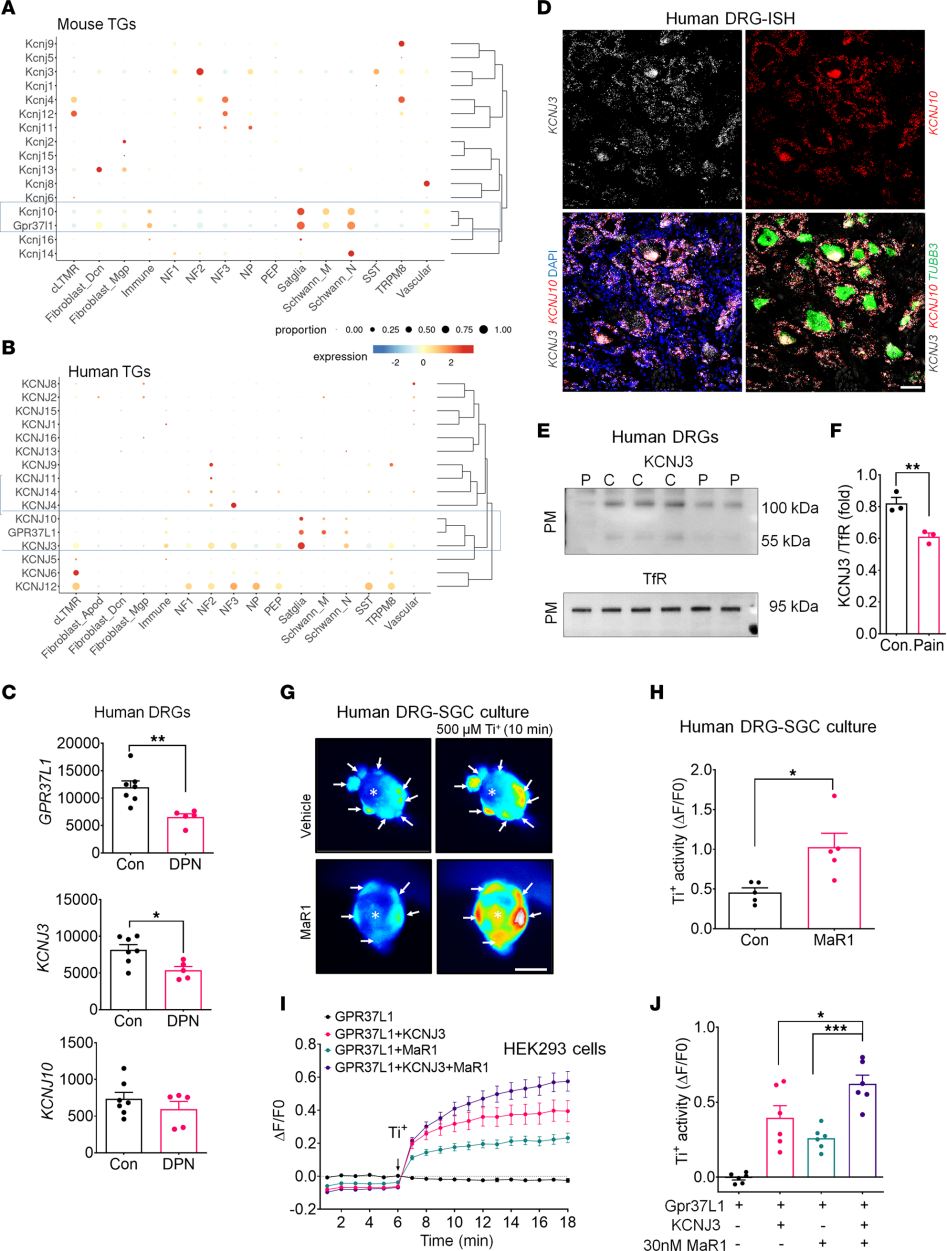
GPR37L1 regulates KCNJ3/GIRK1 signaling in human SGCs. (**A** and **B**) Single-cell RNA-Seq meta-analysis of KCNJ transcript expression in mouse (**A**) and human (**B**) TGs from a published database (36). (**A**) Mouse SGCs predominantly express *Gpr37l1* and *Kcnj10*, which are clustered together. (**B**) Human SGCs express *GPR37L1,* *KCNJ3*, and *KCNJ10*, which are clustered together. Note greater expression of *KCNJ3* than *KCNJ10*. (**C**) Transcriptomic meta-analysis of human DRGs, from a published database (29), reveals downregulations of *GPR37L1* and *KCNJ3*, but not *KCNJ10*, in patients with painful DPN. (**D**) Triple RNAscope ISH showing the expression of *KCNJ10* (red), *KCNJ3* mRNA (white), and *TUBB3* (green*)* in nondiseased human DRGs. Note colocalization of *KCNJ10* and *KCNJ3* in SGCs surrounding the *TUBB3*^+^ neurons. Scale bar: 50 μm. (**E**) Western blots showing PM fractions of KCNJ3 levels in human DRGs of neuropathic pain patients and controls from parallel gels. (**F**) Quantification of the Western blot results in **E** (*n* = 4). (**G** and **H**) MaR1 increases K^+^ levels in human SGCs. (**G**) Images of Ti^+^ influx in human DRG cocultures for SGCs and neurons treated with vehicle and MaR1. SGCs are indicated with white arrows. *: neuron, 20x magnification. Scale bar: 50 μm. (**H**) Quantification of fluorescence intensity for **G** at 10 minutes after 500 μM Ti^+^ simulation in human SGCs. *n* = 5 cultures from 2 donors. (**I**) Time course of Ti^+^ influx in HEK293 cells expressing GPR37L1 with vehicle (*n* = 16) or MaR1 (100 nM, 30 minutes *n* = 16) and GPR37L1/KCNJ3 in the presence of vehicle (*n* = 13) and MaR1 (100 nM, 30 minutes, *n* = 12). (**J**) Quantification of Ti^+^ influx in **I** after 10 minutes of 500 μM Ti^+^ stimulation (*n* = 6 culture). Data are expressed as means ± SEM and were analyzed by 2-tailed *t* test (**C**, **F**, and **H**) or 1-way ANOVA with Tukey’s post hoc test (**J**). *P < 0.05; **P < 0.01; ***P < 0.001.

We used a published database of scRNA-Seq of mouse and human TGs (36, 37) to compare the species differences in expression levels and cell types of the genes encoding the KCNJ family of K^+^ channels in SGCs of mouse TGs (Figure 7A) and human TGs (Figure 7B). In mouse TGs, *Kcnj10* is predominantly expressed by SGCs, although Schwann cells and immune cells show moderate levels of *Kcnj10* expression (Figure 7A). Compared with the mouse gene, human *KCNJ10* expression is lower, but still specific for SGCs (Figure 7B). We found a striking species difference in KCNJ3 expression in mouse and human TGs. *Kcnj3* is expressed by sensory neurons, but not by SGCs in mouse TGs (Figure 7A). In sharp contrast, *KCNJ3* is highly expressed by SGCs, with some low expression in sensory neurons and Schwann cells in human TGs (Figure 7B). Cluster analysis (36) showed that *Gpr37l1* is associated with *Kcnj10* in mouse TGs and *GPR37L1* is associated with both *KCNJ3* and *KCNJ10* in human TGs (Figure 7, A and B). Additional analysis of mRNA expression of human DRGs of DPN and control patients from a published database (29) revealed significant downregulation in the mRNA expression levels of *GPR37L1* (*P* < 0.01) and *KCNJ3* (*P* < 0.05), but not *KCNJ10*, in the DPN group (Figure 7C). ISH analysis showed that both *KCNJ3* and *KCNJ10* are expressed by SGCs surrounding *TUBB3*^+^ neurons in human DRG sections (Figure 7D). Further analysis of human RNA-Seq data (36) revealed a higher percentage of colocalization for *GPR37L1*/*KCNJ3* (~75%) than *GPR37L1*/*KCNJ10* (~30%) (Supplemental Figure 8A). Western blot analysis showed a significant decrease of KCNJ3 in the PM of DRG samples from neuropathic pain patients (*P* < 0.01, versus control, Figure 7, E and F).

The KCNJ3 K^+^ channel is known to be modulated by GPCRs, and the Ti^+^ influx assay has been used for G protein (βγ subunit mediated) drug screening (38). To investigate whether GPR37L1 regulates K^+^ influx via KCNJ3, we conducted a Ti^+^ influx assay in both human SGCs (Figure 7, G and H) and HEK293 cells expressing GPR37L1 or GPR37L1 together with KCNJ3 (Figure 7I). Human DRG culture imaging revealed an increased dye signal in SGCs surrounding neurons following 0.5 mM Ti^+^ incubation. However, MaR1 treatment (100 nM, 0.5 hours) caused significant increases in K^+^ signaling in human SGCs (*P* < 0.05, versus control, Figure 7, G and H, and Supplemental Figure 8B). In GPR37L1/KCNJ3-expressing HEK293 cells, MaR1 treatment was sufficient to increase K^+^ levels (Figure 7I). As expected, KCNJ3 overexpression was also able to increase K^+^ levels in GPR37L1-expressed cells. However, MaR1 caused a further increase in K^+^ levels in KCNJ3/GPR37L1-expressing cells (Figure 7, I and J). Finally, MaR1-induced K^+^ increase in KCNJ3/GPR37L1-expressing cells was blocked by either 1 μM of KCNJ3 antagonist SCH 23390 (39) or 1 μM of G_βγ_ inhibitor (gallein) (40) (Supplemental Figure 8, C and D). MaR1 has no effect on KCNJ3-only–expressing cells (Supplemental Figure 8, E and F).

### GPR37L1 mutations are associated with chronic pain in humans.

**Figure 8:**
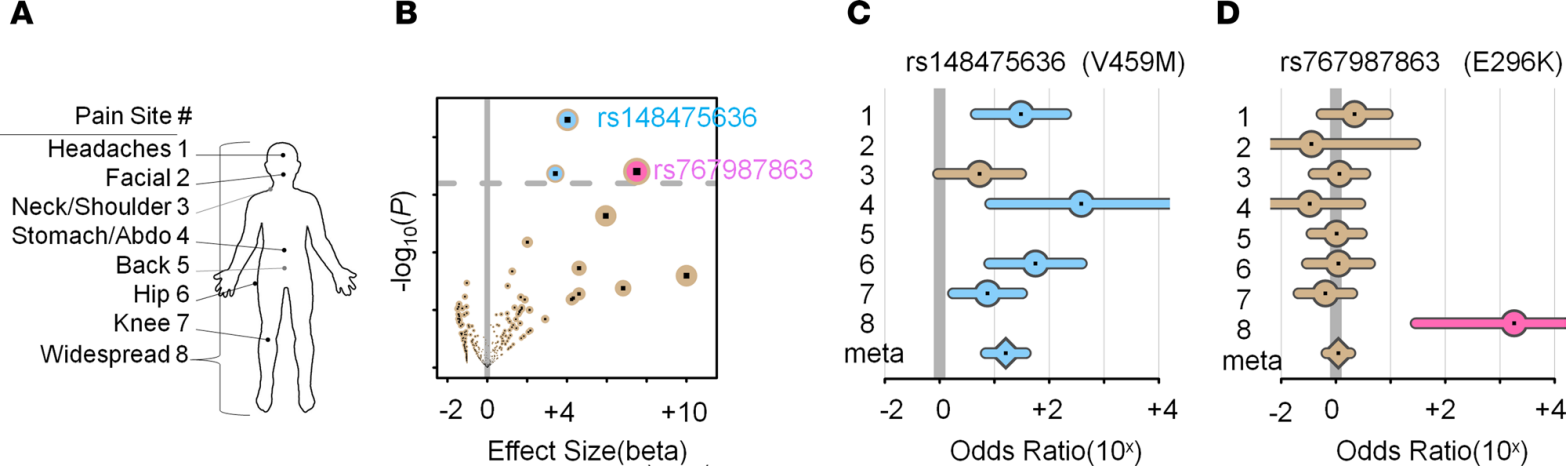
*GPR37L1* rare variant effects in chronic pain. (**A**) The 8 chronic pain sites found in the UK Biobank project. Abdo, abdomen. (**B**) Volcano plot showing rare variants’ significance as a function of effect size. Each dot represents a variant. The vertical bar indicates the null effect, while the dotted horizontal bar indicates the threshold for an FDR of 20%. Two significant variants were identified: rs148475636 (blue) and rs767987863 (pink). (**C**) Forest plot for variant rs148475636. (**D**) Forest plot for variant rs767987863. The forest plots show variants’ effects at the 8 chronic pain sites. Segments track a 95% CI for point estimates of odds ratios. Estimates are not provided for allele counts of less than 5. Meta estimate from a meta-analysis of all available pain sites. Segments are colored (blue/pink) when *P* < 0.05.

We examined the genetic correlation of *GPR37L1* variants with chronic pain. We assessed the UK Biobank cohort to inquire about the potential roles of rare GPR37L1 variants in chronic pain, as it offered accurate allele assignments via whole-exome sequencing (41). SAIGE was used to perform association tests between rare coding variants and a report of chronic pain at 8 body sites (Figure 8A and Supplemental Table 3A). The rare coding variants were chosen for analyses because they are most likely to have a significant and interpretable effect and they usually result in a reduction of the function of the corresponding protein rather than an increase. Furthermore, they provide a precise target for follow-up functional analysis (42). A total of 380 variant-site associations with at least 5 minor allele counts were considered in a primary analysis (Figure 8B). Of these, 3 were found to be significant at the FDR level of 20%: rs148475636 (V459M) was associated with hip pain and headache (OR = 56.1, *P* = 5.0 × 10^–5^, and OR = 30.5, *P* = 3.95 × 10^–4^) and rs767987863 (E296K) was associated with widespread pain (OR = 1816, *P* = 3.9 × 10^–4^) (Supplemental Table 3A). We then performed secondary analyses to estimate the risk for chronic pain at all 8 sites for 2 identified variants (Figure 8, A–D). The variant of SNP rs148475636 was associated with significantly increased risk at many body sites, suggesting a pleiotropic effect, including an overall significant and large risk in a meta-analysis (meta OR = 16, *P* = 1.0 × 10^–10^), while a meta-analysis on SNP rs767987863 was not significant (Figure 8, A and D, and Supplemental Table 3B). Importantly, both significant SNPs are nonsynonymous and display high CADD scores (43), suggesting that the variant alleles are deleterious and have a loss of function (8.0 for rs148475636 and 27.3 for rs767987863; Supplemental Table 3A). More broadly, the majority of the tested coding variants had substantial pathogenicity CADD scores (Supplemental Table 3A) and were risk factors for chronic pain (Figure 8D), suggesting that GPR37L1 is protective against pain. Further, in silico prediction of protein stability revealed that, among the 6 mutations of *GPR37L1* variants, the allele E296K showed a significant loss of protein stability (*n* = 9 simulations, Supplemental Figure 9A).

Finally, we investigated the impact of E296K mutation on GPR37L1 expression and function. Overexpression of both WT and E296K mutant *GPR37L1* in human SGCs resulted in significant increases of *GPR37L1* mRNA levels compared with mock-transfected cells (*P* < 0.01) without altering *KCNJ3* mRNA expression (Supplemental Figure 9B). However, no significant difference in PM expression of GPR37L1 was observed between WT and mutant groups in HEK293 cells (*P* = 0.10, Supplemental Figure 9, C–E). We also conducted functional assays in human SGCs expressing WT and mutant *GPR37L1*. We found significant increases in IL-1β production in mutant-expressing SGCs with PTX treatment (*P* < 0.01, Supplemental Figure 9F). Moreover, MaR1 increased Ti^+^ influx activity in WT, not E296K mutant, expressing cells (Supplemental Figure 9G). Collectively, these findings suggest that the E296K mutation may impair the function of GPR37L1.

## Discussion

In this study, we have offered several insights into GPR37L1 and satellite glial signaling in pain control. First, we demonstrated a protective role of GPR37L1 against chronic pain, as neuropathic pain caused by chemotherapy or STZ-induced pain is enhanced and prolonged in transgenic mice with *Gpr37l1* deficiency (*Gpr37l1^+/–^* and *Gpr37l1^–/–^*). We also showed that partial knockdown of GPR37L1 in DRGs of naive WT animals was sufficient to induce mechanical allodynia. Furthermore, we found GPR37L1 downregulation not only in DRGs of mice with chemotherapy and diabetes, but also in DRGs of patients with painful diabetic neuropathy. Importantly, we have identified SNPs of human *GPR37L1* and potential variant alleles that are deleterious with loss of function, supporting our functional studies in mice showing that GPR37L1 is protective against the development of chronic pain.

Another finding is that we identified MaR1 as a ligand of GPR37L1. Prosaposin, a highly conserved glycoprotein and secretory protein, was initially implicated as a ligand for GPR37 and GPR37L1 (10). Single-cell–based pathway analysis in mouse DRGs also revealed an association of prosaposin and *Gpr37l1* (44). However, it is unclear how prosaposin interacts with GPR37L1. Our data showed that *Gpr37l1* and *GPR37L1* transcripts are among the top 10 expressed GPCR transcripts in mouse and human DRGs. Given its high expression under physiological conditions, constitutive activity of GPR37L1 was proposed, and metalloprotease cleavage of the N terminal of GPR37L1 may reduce its constitutive activity (12). It was also suggested that this orphan receptor remains unliganded (13). MaR1 is a DHA-derived SPM that has exhibited potent analgesic actions in animal models of inflammatory pain (24), arthritic pain (26), and postoperative pain (45). So far, all the known SPM receptors are GPCRs and each SPM may have multiple receptors (23). It was found that the GPCR LRG6 acts as a specific receptor for MaR1, and activation of LRG6 by MaR1 promoted phagocyte immune-resolvent functions (46). Notably, LRG6 was identified by β-arrestin assay, and GPR37L1 showed no such activity (46). Our data showed a specific signaling of MaR1 through GPR37L1. The lipid overlay and protein pull-down experiments demonstrated MaR1 binding to GPR37L1. Furthermore, the computer simulations revealed that MaR1 forms hydrogen bonding with ASN190, ARG196, and GLU375 residues in the GPR37L1-binding pocket, whereas NPD1 only showed weak binding to this receptor. Importantly, our functional evaluations demonstrated that GPR37L1 is required for MaR1’s analgesic actions in mouse models of neuropathic pain.

Our results demonstrate that GPR37L1 controls neuropathic pain in mice by regulating the surface expression and function of KCNJ10 (Kir4.1) in SGCs. We have provided a previously underappreciated mechanism by which chemotherapy and diabetes induce pain via SGC signaling. Chemotherapy caused a rapid suppression of KCNJ10-mediated K^+^ currents in SGCs. Remarkably, MaR1 conferred protection against chemotherapy-induced peripheral neuropathy (CIPN) by inducing GPR37L1-dependent upregulation of surface KCNJ10 expression and KCNJ10-mediated K^+^ currents in SGCs. We postulate that dysregulation of GPR37L1/KCNJ10 signaling in SGCs in neuropathy conditions (CIPN and DPN) will impair the SGC’s function of K^+^ buffering, leading to sequential increases in extracellular levels of K^+^, nociceptor excitability, and pain sensitivity. Importantly, this dysregulation can be prevented and treated by GPR37L1 agonists such as MaR1 (Supplemental Figure 10). Additionally, MaR1 also blocked PTX-induced IL-1β release in SGC-neuron cocultures. Control of neuroinflammation in DRG by the MaR1/GPR37L1 axis will further reduce neuropathic pain.

Development of pain medicine has been hampered by the translational gap between rodents and humans, as many genes are differentially expressed and functioned in mouse and human tissues including DRGs (47–50). We validated the critical role of the MaR1/GPR37L1 axis in both mouse (SGC) and human cells (SGC and HEK cells). Both *Gpr37l1* and *GPR37L1* mRNAs are highly expressed in mouse and human DRGs. We also found striking species differences in KCNJ3 and KCNJ10 expression in mouse and human TGs. In mouse sensory ganglia, *Kcnj3* is expressed by neurons, but not by SGCs (Figure 7A) (17, 51). However, in human sensory ganglia, *KCNJ3* mRNA is highly enriched in SGCs (Figure 7B). This distinction highlighted different mechanisms of KCNJ3 in pain modulation in mice and humans. As an inwardly rectifying potassium channel (GIRK), KCNJ3 (GIRK1) regulates the G protein–mediated outward K^+^ currents in neurons induced by analgesics, such as opioids (52). Importantly, KCNJ3 is coupled to GPR37L1 in human SGCs and its activity is positively regulated by MaR1. Although KCNJ10 (Kir4.1) is expressed by SGCs of both mouse and human sensory ganglia, its contribution to K^+^ buffering could be greater in mice than humans. In mouse sensory ganglia, GPR37L1 is only associated with KCNJ10. However, in human sensory ganglia, GPR37L1 is associated with both KCNJ10 and KCNJ3, with much higher colocalization with KCNJ3 than KCNJ10 (Supplemental Figure 8A). Despite these species’ differences, the MaR1/GPR37L1 pathways are critical for SGC K^+^ signaling in both mice and humans.

Neuropathic pain after DPN and CIPN is a major health problem (53–55). Current treatments for neuropathic pain are insufficient and cause significant side effects (56, 57). Our findings suggest that targeting GPR37L1 in SGCs will lead to new therapeutics for treating neuropathic pain after CIPN and DPN. MaR1 is a highly potent GPR37L1 activator and has a wide safety profile, but its pharmacokinetics and in vivo stability (e.g., short half-life as a lipid mediator) remain to be improved. An alternative is to identify small-molecule agonists of GPR37L1 for pain management. Although we have shown specific and high expression of GPR37L1 in mouse and human SGCs, astrocytes in the central nervous system also express GPR37L1, which plays a protective role in astrocytes (10). The study of GPR37L1’s function and its activation by MaR1 in astrocytes is of great interest. Notably, SGCs and astrocytes reside in the PNS and CNS, respectively, and play similar and distinct roles in pain regulation (6). Given the well-known side effects of CNS-targeting drugs, targeting SGC-GPR37L1 in the PNS may offer a safer analgesic drug for pain relief and disease modification. Notably, the DRG sensory neuron has a single process (the stem axon) that bifurcates into a peripheral and a central axonal branch. Thus, action potential firing may be affected by the T-junction in DRG neurons (58). Since GPR37L1 is also expressed by Schwann cells in the peripheral nerve, future studies are needed to investigate how GPR37L1 in SGCs or Schwann cells directly or indirectly regulates action potential firing in cell bodies and axons.

## Methods

### Sex as a biological variable.

Both male and female animals were used in this study. The numbers of male and female animals used in different experiments are described in Supplemental Table 4. Human DRG donors of both sexes were used as described in Supplemental Table 5.

### Animals.

The *B6;129S5-Gpr37l1^tm1Lex^/Mmucd* strain was obtained from UC Davis (MMRRC, stock 011709-UCD). *Gpr37l1* and littermate mice with a C57BL/6 background were maintained at Duke University Medical Center. CD1 mice were also used for some behavioral, histochemical, and electrophysiological studies. Adult mice (males and females, 8–10 weeks) were used for behavioral tests and biochemical assays. Both sexes were included in behavioral testing, and no sex differences were noticed in behavioral and cellular tests conducted in this study. Two to 5 mice were housed in each cage under a 12-hour light/12-hour dark cycle with ad libitum access to food and water. Sample sizes were estimated based on our previous studies for similar types of behavioral and biochemical analyses (28).

### Pain models and drug injection.

Mouse models of CIPN were induced by PTX given via i.p. route either by a single injection or by 4 injections. A mouse model of DPN was induced by STZ via an i.p. injection. For i.t. injection of MaR1 or PTX, a spinal cord puncture was made by a Hamilton micro-syringe between the L5 and L6 levels. See more details in Supplemental Methods.

### I.g. injection.

Mice were placed in a prone position under isoflurane anesthesia, and microinjection of 1–2 μl of the solution of siRNA, PTX, MaR1, and AAV-virus was administrated to L4 and L5 DRG using a Hamilton syringe connected to a glass micropipette. See Supplemental Methods for additional details.

### Reagents.

MaR1 (catalog 10878) and other lipid mediators were purchased from Cayman Chemical. See Supplemental Methods for all reagents.

### Lipid overlay assay.

Lipid membrane coating and protein overlay assay were performed as previously described (59). Lipid mediators, including SPMs (RvD1, RvD2, RvD3, NPD1, MaR1), DHA, and vehicle EtOH, were directly loaded on hydrophobic PVDF membrane walls (96-well plates; Bio-Rad). Compound-coated membranes were dried and blocked with 1% BSA. The coated membranes were incubated with lysates obtained from *hGPR37L1-*transfected HEK293 cells or with DRG lysates from WT or *Gpr37l1*-KO mice for 2 hours, followed by detection using an anti-GRP37L1 antibody (Bioss, rabbit, 1:1000, catalog bs-15390R) or anti-flag antibody (Cell Signaling Technology, rabbit, 1:1000, catalog14793). Blots were further incubated with an HRP-conjugated secondary antibody (anti-rabbit, Jackson ImmunoResearch, raised in donkey, 1:5,000), developed in ECL solution (Pierce), and protein signal was visualized using ChemiDoc MP (Bio-Rad). The signal intensity was quantified by ImageJ software (NIH).

### Co-IP.

Mouse DRGs were harvested, washed once in ice-cold PBS, and then scraped in harvest buffer (10 mM HEPES, 100 mM NaCl, 5 mM EDTA, 1 mM benzamidine, protease inhibitor tablet, 1% Triton X-100, pH 7.4). Cell lysates were then solubilized and immunoprecipitated with anti-GPR37L1 antibody (Bioss, rabbit, 1:100, catalog bs-15390R), anti-Kir4.1/KCNJ10 antibody (Thermo Scientific, rabbit, 1:100, catalog 12503-1-AP), or normal rabbit IgG (Santa Cruz Biotechnology Inc., 1:100, catalog sc-2027). The antibody-binding proteins were pulled down using protein A–conjugated magnetic beads (Thermo Scientific, catalog88845) and washed by repeated centrifugation and homogenization. Samples were heated, then probed by Western blotting using anti-GPR37L1 (Alomone Labs, rabbit, 1:1000, catalog AGR-050) or anti-Kir4.1 (Thermo Scientific, guinea pig, 1:1000, catalog PA5-111798).

### Human DRG samples.

Nondiseased human DRGs were obtained from donors. Human DRGs from neuropathic pain patients and healthy controls were obtained from a cohort collected at the University of Pittsburg (60). They consisted of a snap-frozen bilateral lumbar L4 and L5 collected from brain-dead subjects following asystole with the consent of first-tier family members.

### Human DRG SGC-neuron cocultures.

Human DRG cultures were prepared as previously reported. DRGs were digested at 37°C in a humidified CO_2_ incubator for 120 minutes with collagenase type II (Worthington, 290 units/mg, 12 mg/ml final concentration) and dispase II (Roche, 1 unit/mg, 20 mg/mL) in PBS with 10 mM HEPES, pH adjusted to 7.4 with NaOH. DRGs were mechanically dissociated using fire-polished pipettes, filtered through a 100 µm nylon mesh, and centrifuged (500*g* for 5 minutes). The pellet was resuspended, plated on 0.5 mg/mL poly-d-lysine–coated glass coverslips, and grown in Neurobasal Medium (Thermo Fisher Scientific) supplemented with 10% FBS, 2% B-27 supplement, and 1% penicillin/streptomycin. K^+^ influx assay was performed after 7 days in culture. 

### Western blot.

Protein samples were prepared from transfected cells, lipid pull-down beads, and DRGs. Tissue and cells were placed on ice and lysed with ice-cold RIPA. See more details in Supplemental Methods.

### ISH using RNAscope probes.

Mice were transcranial perfused with PBS followed by 4% PFA under deep anesthesia with isoflurane. Lumbar DRGs, TG, and nodose ganglia were isolated and post-fixed in the same fixative. Tissues were cryopreserved in a sucrose gradient, then embedded in OCT medium (Tissue-Tek) and cryosectioned at 14 μm. The RNAscope probes against mouse *Gpr37l1* (catalog 319301), mouse *Gpr37* (catalog 319291), human *GPR37L1* (catalog 513641), human *KCNJ3* (catalog 1154801-C3), human *KCNJ10* (catalog 461091), and human *TUBB3* (catalog 481251) were designed by Advanced Cell Diagnostics, and the RNAscope multiplex fluorescent assays were conducted according to the manufacturer’s instructions. Prehybridization and hybridization were performed according to standard methods (34).

### IHC.

Mice were deeply anesthetized with isoflurane and perfused through the ascending aorta with PBS, followed by 4% PFA. After the perfusion, L4–L5 DRGs were removed from the mice and post-fixed in the same fixative overnight. Human DRGs were obtained from the NDRI and fixed in 4% PFA overnight. See more details in Supplemental Methods.

### Patch-clamp recordings in mouse SGCs in whole-mount DRG preparation.

Under urethane anesthesia, mice were rapidly euthanized and lumbar DRGs were dissected and briefly digested. SGCs in whole mouse DRG could be visualized using a ×40 water-immersion objective on an Olympus BX51WI microscope. A whole-cell patch-clamp configuration was made on SGCs as reported (61). The Kir4.1 currents were obtained by digitally subtracting those currents in the absence and presence of barium (35, 62). See more details in Supplemental Methods.

### Ti^+^ influx assay.

Ti^+^ flux assay was conducted using the manufacturer’s and standard protocol (63). For examining Ti^+^ flux in GPR37L1, KCNJ3, KCNJ3/KCNJ10-expressing Hek293 cells, and WT/E296K-GPR37L1–expressing human SGCs, cells were dislodged from tissue culture flasks. After overnight incubation, the cell culture medium was replaced with thallium influx dye–loading solution. Following a 60-minute incubation at room temperature, data were collected from a plate reader. See more details in Supplemental Methods.

### Nociceptive behavior tests.

*Gpr37l1^+/+^*, *Gpr37l1^+/–^*, or *Gpr37l1^–/–^* mice were habituated to the testing environment for at least 2 days before baseline testing. All animal behaviors were tested blindly. Thermal and mechanical sensitivity was tested before and after the injection of PTX (1 × 6 mg/kg, or 4 × 2 mg/kg, i.p.) or STZ (75 mg/kg, i.p.). MaR1 was i.t. injected 3 days after the STZ or PTX injection. For testing mechanical sensitivity, mice were confined in boxes (14 × 18 × 12 cm) placed on an elevated metal mesh floor, and their hind paws were stimulated with a series of von Frey hairs with logarithmically increasing stiffness (0.16–2.00 g, Stoelting), presented perpendicularly to the central plantar surface. The 50% paw-withdrawal threshold was measured by Dixon’s up-down method (64). Thermal sensitivity was measured using a Hargreaves radiant heat apparatus (65) (IITC Life Science). The basal paw withdrawal latency was adjusted to 10–15 seconds, with a cutoff of 25 seconds to prevent tissue damage. Acetone (50 μl) was applied through wire mesh flooring onto the plantar surface of the infected hind paw to produce evaporative cooling (66). Mechanical and thermal sensitivity were also tested after intra-DRG injection of siRNA and MaR1.

### Genome-wide association.

Genome-wide association tests were performed in the large UK Biobank project cohort comprising half a million participants (41, 67). Information about chronic pain reports collected at the visit was available at 8 body sites: head (headaches), face, neck/shoulder, stomach/abdominal, back, hip, knee, and widespread. Variants were annotated to GRCh38 reference provided by UK Biobank via Resource 3803 with Ensembl Variant Effect Predictor (VEP, release 99) (68). Rare coding variants were tested, and we required a minor allelic frequency of less than 0.01 and a minor allele count greater than 5. We used SAIGE (version 0.44.2) (69) to perform the tests, as it considers cryptic relatedness and guards against false-positive associations in the face of case-to-control imbalance (due to the cross-sectional nature of the cohort) by means of the saddle point approximation method (70). The meta-analysis at a given variant position across all 8 chronic pain sites was performed using the inverse variance-based weighting scheme (71). A combined annotation-dependent depletion score (CADD, version 1.6) (43) was used to estimate the deleteriousness of the variants. See more details in Supplemental Methods.

Additional information regarding the following is available in Supplemental Methods: reagents; genotyping; pain models and drug injection; RNA purification, sequencing, and analysis; HEK293 cell culture and cDNA transfection; lipid pull-down assay; immortal human SGC cell line generation and transfection; mouse SGC cultures and SGC-neuron cocultures; computer simulations; i.g. injection; ELISA assay; flow cytometry; prediction of protein stability; real-time quantitative PCR; preparation of PM fraction, Western blot; IHC; patch-clamp recordings in mouse SGCs; Ti^+^ influx assay; and genome-wide association.

### Statistics.

All data are expressed as means ± SEM. The sample size for each experiment is indicated in the figure legends. GraphPad Prism, version 8.0, software was used to perform statistical analysis. Data were analyzed by 2-way ANOVA, followed by Bonferroni’s post hoc test or Tukey’s post hoc test for multigroup comparisons or by Student’s *t* test for 2-group comparisons. *P* < 0.05 was considered statistically significant.

### Study approval.

All the animal procedures were approved by the Institutional Animal Care and Use Committee of Duke University. Animal experiments were conducted in accordance with the NIH *Guide for the Care and Use of Laboratory Animals* (National Academies Press, 2011). Nondiseased human DRGs were obtained from donors through the National Disease Research Interchange (NDRI) with permission of exemption from the Duke University Institutional Review Board. All procedures were approved by the University of Pittsburgh Committee for Oversight of Research and Clinical Training Involving Decedents and the Center for Organ Recovery and Education, Pittsburgh, Pennsylvania, USA (http://www.core.org).

### Data availability.

No custom software was used in this study. The data and code are available upon request. Next-generation sequencing data have been deposited in the NCBI’s Gene Expression Omnibus database (GSE260669 and GSE78150 for mouse and human DRG-sequencing data, respectively). Supporting data values were included in the source file for the original data. Values for all data points in graphs are reported in the Supporting Data Values file.

## Author contributions

SB and RRJ developed the project. SB, CJ, JX, SC, AM, XL, QH, YL, and ZW conducted experiments and data analyses. XA, MP, LOFDA, and SJE contributed to the genome association study under the supervision of LD. RT, TB, and QZ participated in project development. SB and RRJ wrote the manuscript. LD, TB, and other coauthors edited the manuscript.

## Supplementary Material

Supplemental data

Unedited blot and gel images

Supplemental table 1

Supplemental table 2

Supplemental table 3

Supplemental table 4

Supplemental table 5

Supporting data values

## Version 1 03/26/2024

In-Press Preview

## Version 2 05/01/2024

Electronic publication
