# Supplementary material for: Satellite glial GPR37L1 and its ligand maresin 1 regulate potassium channel signaling and pain homeostasis
Source: J Clin Invest. 2024 Mar 26;134(9):e173537. doi: 10.1172/JCI173537 (PMC11060744; doi:10.1172/JCI173537)
Supplement: Supplemental data [file jci-134-173537-s201.pdf]

Satellite glial GPR37L1 and its ligand maresin 1 regulate potassium channel signaling and pain homeostasis

Sangsu Bang, Changyu Jiang, Jing Xu, Sharat Chandra, Aidan McGinnis, Xin Luo, Qianru He, Yize Li[1], Zilong Wang, Xiang Ao, Marc Parisien, Lorenna Oliveira Fernandes de Araujo, Sahel Jahangiri Esfahan, Qin Zhang, Raquel Tonello, Temugin Berta, Luda Diatchenko, and Ru-Rong Ji

Supplemental Information

Supplemental Methods with References

Supplemental Figures (1-10)

Supplemental Tables List (1-5)

Supplemental Methods with References

Reagents

Maresin 1 (MaR1, Cat No. 10878) and other lipid mediators were purchased from Cayman Chemical. *GPR37L1*-Tango plasmid was from Addgene (Cat No.66356). Human *GPR37L1-WT* and human *GPR37L1-E296K* mutants were synthesized from Twist Bioscience (South San Francisco, CA, US). The human *KCNJ10-v5* plasmid was from DNASU (Clone: HsCD00939635). Human *KCNJ3-HA* plasmid was purchased from Applied Biological Materials (Richmond, BC, Canada; Cat no: 253840610295). *Gpr37l1* siRNA and control scRNA were purchased from Ambion life technology (Cat No. 173005 for *Gpr37l1*, Cat No, 4459405 for *scRNA*), and transfection agent PEI/RNA polyplexes (LNP102) were from ABP bioscience (Cat No. LP007-1; Rockville, MD). Flag-tagged *human GPR37L1* was purchased from Origene (Rockville, MD, Cat No. RC208132). *Fabp7-mGpr37*-AAV*9* or mock AAV9 virus was generated by Vector Builder (Chicago, IL). The Brilliant Thallium flex kit (Cat No. 11000-10) was purchased from Ion Bioscience (San Marcos, TX). Paclitaxel (T719) and streptozotocin (S0130) were purchased from Sigma-Aldrich. SCH 23390 (0925), ML297 (5380), and Gallein (3090) were purchased from Tocris (Minneapolis, MN).

Genotyping for *Gpr37l1* mutant mice

Because of the proximity of PCR bands of WT and KO genes, we used two separate pairs of primers to characterize WT (+/+), heterozygous (+/-), and KO/homozygous (-/-) mice.

WT-pair (385 bp): Primer 1-0579-24 & Primer 2-0579-5

Mutant pair (366 bp): Primer 1-0579-24 & Primer 3-Neo3a

Primer-0579-24: *ACACCTGCCTGTTCATCTGG*

Primer-0579-5: *CACAGCTACTACTTGAAGAG*

Primer-Neo3a: *GCAGCGCATCGCCTTCTATC*

HEK293 cell culture and transfection of *GRP37L1, KCCNJ10,* and *KCNJ3* cDNAs.

The HEK293 Flp-In^{TM} cell line (Invitrogen, R78007) was purchased from the Duke Cell Culture Facility. Cells were cultured in high glucose (4.5 g/L) Dulbecco's Modified Eagle's Medium containing 10% (v/v) fetal bovine serum (Gibco). The *GPR37L1*, *GPR37L1-WT*, *GPR37L1-E296K*, *Mock*, *KCNJ10*, and

KCNJ3 cDNAs were transfected using Lonza 4D-Nucleofector™ X-unit (2 µg cDNA/1 x 10^7 cells; protocol No.CM130). After reaching 70% confluency by co-transfection with 1 µg of pmaxGFP control cDNA (Lonza), the transfected cells were cultured for an additional 48 h before use.

Human SGC/neuron-rich culture

Human DRG cultures were prepared as previously reported (1). DRGs were digested at 37°C in a humidified CO2 incubator for 120 min with collagenase Type II (Worthington, 290 units/mg, 12 mg/ml final concentration) and Dispase II (Roche, 1 unit/mg, 20 mg/mL) in PBS with 10 mM HEPES, pH adjusted to 7.4 with NaOH. DRGs were mechanically dissociated using fire-polished pipettes, filtered through a 100-m nylon mesh, and centrifuged (500 g for 5 min). The pellet was resuspended, plated on 0.5 mg/mL poly-D-lysine–coated glass coverslips, and grown in Neurobasal medium supplemented with 10% FBS, 2% B-27 supplement, and 1% penicillin/streptomycin. After 7 days, we performed a K^+ influx assay.

Pain models and drug injection

Mouse models of chemotherapy-induced peripheral neuropathy (CIPN) were induced by paclitaxel, given via intraperitoneal route either by a single injection (6 mg/ml, i.p.) or by 4 injections (2 mg/kg, i.p.) on days 0, 2, 4, 6 (2). A mouse model of diabetic peripheral neuropathy (DPN) was induced by streptozotocin (STZ) via an intraperitoneal injection (75 mg/kg) (3). For intrathecal (i.t.) injection of MaR1 or PTX, a spinal cord puncture was made by a Hamilton micro-syringe (Hamilton) with a 30-gauge needle between the L5 and L6 levels to deliver reagents (5 µl) to the cerebral spinal fluid (4).

RNA purification, sequencing, and analysis

To perform poly-A RNA-sequencing, L1-L5 mouse DRG was homogenized with an RNeasy Mini Kit (Qiagen, Valencia, CA) using on-column DNase-I digestion according to the manufacturer’s protocol. RNA quality was assessed using a Nano-drop (Thermo Scientific) and RNA sequencing service was requested from LC science (Houston, TX). For the mouse DRG RNA-seq analysis, paired-end sequencing was performed with a read length of 150 bp. Read counts per gene per sample were quantified using HTSeq version 0.9.1. The detected total gene number is 25.091–25.664 in DRG samples. GPCR mRNA expression levels in human DRGs were obtained from the human DRG expression quantitative trait loci study (5). In short, DRG expression levels were determined from total RNA extracts measured using

Affymetrix' Human Transcriptome Array 2.0 (Affymetrix, Santa Clara), scanned with Affymetrix' GeneChip 3000 G7 Instrument System, then probe intensities were normalized in a standard fashion.

Lipid pull-down assay

Isolated membrane proteins were pre-adsorbed to uncoated control agarose beads (Vector labs); the unbound fraction was collected and incubated for 24 h at 4°C with lipid-coated agarose beads (6). After extensive washing, bound proteins were eluted from the lipid-coated beads, suspended in Laemmli buffer containing 2% SDS (w/v) and 0.3M β-mercaptoethanol (Sigma), and heated for 5 min at 95°C to dissolve proteins before separation on 4–20% polyacrylamide/SDS gels. The lysate proteins were detected by western blot.

Immortal human SGCs cell line generation and transfection

We stumbled upon cells proliferating beyond passage 12 that were discovered by chance after primary culture from a DRG donor (NDRI, Philadelphia, PA). WT or *mutant GPR37L1* were transfected to SGCs cell line using TransIT®-LT1 reagent (Mirus Madison, WI, USA, Cat Bo.MR2300). The control and mutant GPR37L1 or KCNJ3 expression levels were tested by Real-time PCR method.

Mouse SGC cultures and SGC-neuron co-cultures

DRGs were collected from both WT and KO mice (8 weeks male) and were incubated in 1 mg/ml Collagenase/Dispase (Roche Diagnostics, Madison, WI, USA) at 37°C for 60 min, with agitation at 100 RPM and then followed by incubation in 0.05% trypsin/EDTA for 10 min. The digestion enzymes were prepared in Dulbecco's modified Eagle medium/F12 with GlutaMAX (ThermoFisher). After incubation with 0.1% trypsin inhibitor and centrifugation (300G), the cell pellet was gently triturated in a neurobasal medium containing 0.5 μM glutamine. Dissociated DRG cells were seeded on non-coated culture dishes for 4 h. SGCs and neurons were included for co-culture by hand-shaking of the culture flasks gently for 5 to 10 min and then resuspended by replacing them with a new neurobasal medium. The attached glial-like cells were cultured in the DMEM/F12 medium containing 10% fetal bovine serum and 1% streptomycin/penicillin to promote cell growth and inhibition of differentiation. After 2 to 6 days in culture, the glial-like cells were differentiated by application of the serum-free neural basal medium. The neuron-rich fraction was seeded on a Poly L lysine (Sigma) coated plate with a Neurobasal medium containing 2% B27 (ThermoFisher) (7).

Computer simulations

The protein sequence of human GPR37L1 was downloaded from the UniProt database (ID: O60883) in *fasta* format. The predicted topology for GPR37L1 in UniProt was used for seven-transmembrane alignments and long-loop identification. Template selection and homology modeling were performed using the automated modeling server GPCR-ModSim (8). Human EDNRB (PDB: 6IGK) was selected as a template and active conformation of the model was generated. All other loops were also refined by the Prime module. Ligands were drawn in the Maestro suite in 2D format and were structurally preprocessed using LigPrep from the Schrodinger Suite (Schrödinger Release 2018-4: LigPrep, Schrödinger, LLC, New York, NY, 2018.). Protonation at a physiological pH (7±2) and energy minimization were performed using the OPLS3 force field. To elucidate the binding mode of all ligands in the binding site of the homology model of hGPR37L1, docking studies were performed with the help of Autodock4 software (9). Before the docking, the hGPR37L1 structure was prepared using the AutoDock Tools 4 software. The stability and intra-molecular conformational changes of the protein and molecular dynamics simulations (MDS) were performed on a 1000 ns time scale for the protein-ligand complex and 1000 trajectory structures were recorded. Using the GPU-accelerated DESMOND software (10), the top-scored docking poses were subjected to solvent-explicit, all-atom MDS. The OPLS-2005 force field was used for model generation of the protein-ligand complex, energy minimization, and MDS. The protein was inserted into the POPC lipid bilayer and the full system was immersed in a periodic orthorhombic water box TIP3P. The NPT ensemble class was used with the temperature set to 300 K and pressure set to 1.01325 bar. The trajectory clustering method of Desmond was used to cluster 1000 trajectory structures into ten clusters based on atomic root mean square deviation (RMSD). Then 100 ns MDS was performed on the cluster 1 structure to assess the stability of the docking complex. 2D structure was plotted using protter software.

Intraganglionic injection of siRNA, PTX, MaR1, and AAV-virus

Mice were placed in a prone position under isoflurane anesthesia and a 1 cm posterior longitudinal skin incision was made in the lumbar portion of the spine. After the plate of the ipsilateral L5 vertebra was carefully removed to expose the L5 spinal nerve and the L4-L5 DRGs, microinjection of 1-2 µl of the solution was administrated to L4 and L5 DRG using a Hamilton syringe (80030) connected to a glass micropipette (tip diameter 10–20 μm) (11). After injection, the wound was covered with a gelatin sponge solution to prevent leakage and then closed with a suture. The following reagents were injected with gelatin sponge. siRNA: 3 μg of *Gpr37l1*-targeting siRNA or scramble RNA was mixed with PEI/RNA

polyplexes (1 µl LNP102). MaR1 (100 ng) or PTX (100 ng) with PBS as a vehicle was injected with gelatin spongey (4 µl). AAV9-*Fabp7-mouse Gpr37L1* (1 x 10^{12}) were injected (2 µl) with gelatin spongey.

ELISA assay

Mouse IL-1β levels were measured using a mouse IL-1β ELISA Kit (R&D, Cat No. MLB00C, Minneapolis, MN), Human IL1β were detected using a human IL-1 β ELISA Kit (Proteintech, Rosemont, IL; Cat No. KE00021) according to the manufacturer's instructions. Briefly, 100 µl of culture medium was added to each well and a standard curve was included for each experiment.

Flow cytometry

Mouse DRG tissues were dissociated with 1 mg/ml Collagenase/Dispase (Roche) in a shaking incubator for 90 min. The dissociated tissues were incubated in 10% FBS-supplemented DMEM media for 1 h for neutralizing of the enzymes. The dissociated cells were washed out using a PBS + 10 mM EDTA solution. The cells were then fixed with 2% paraformaldehyde (PFA) and permeabilized with HBSS + 2% triton X-100. All dissociated cells were blocked with Fc receptors staining buffer (1% anti-mouse CD16/CD32, 2.4 G2, 2% FBS, 5% NRS, and 2% NMS in HBSS; BD Bioscience) and then stained with a standard panel of antibodies: Glast-PE (rat-IgG, Cat No.130-118-483, 1:200, Miltenyl Biotech), Nissle-cy5 (Sigma, 1 µg/ml), and GPR37L1-Apc-cy7 (rabbit IgG, Cat No. bs-15390R, Bioss, 1 µg/ml). After staining, cells were washed in PBS with EDTA. The flow cytometry events were acquired in a BD FACS Canto II flow cytometer by using BD FACS Diva 8 software (BD Bioscience). Data were analyzed using Cytobank Software (https://www.cytobank.org/cytobank).

Prediction of protein stability

To predict the stability of the protein in mutant GPR37L1, we used web-based protein stability software in known or newly discovered mutations. Change instability from a mutant (M) protein to a wild-type (W) form was defined as the difference in the corresponding unwinding free energies ΔΔG of the two proteins. A negative ΔΔG value indicates that the mutant protein is more unstable. To calculate the protein stability, we used 9 different web-based prediction servers and presented the ΔΔG value (12, 13). The prediction servers are as follows:

PremPS: https://lilab.jysw.suda.edu.cn/research/PremPS/

DDgun: https://folding.biofold.org/ddgun/index.html#!

Mupro: http://mupro.proteomics.ics.uci.edu/

SAAFEC-SEQ: http://compbio.clemson.edu/SAAFEC-SEQ/

I-mutant: https://folding.biofold.org/cgi-bin/i-mutant2.0.cgi

DUET: http://biosig.unimelb.edu.au/duet/stability

DeepDDG: http://protein.org.cn/ddg.html

mCSM: http://structure.bioc.cam.ac.uk/mcsm

SDM: http://www-cryst.bioc.cam.ac.uk/~sdm/sdm.php

Real-time quantitative PCR

Real-time quantitative PCR (RT-qPCR) assays were conducted in cDNA samples obtained from a reverse-transcription reaction, using the BioRad CFX96 system (BioRad). Total RNA from the DRG and SGC cultures was extracted using the Direct-zol RNA Miniprep Kit (Zymo Research, Irvine, CA), and 0.5–1 μg RNAs were reverse transcribed using the iScript cDNA Synthesis Kit (Bio-Rad). Specific primers, including the GAPDH control, were designed using IDT SciTools Real-Time PCR software. We performed gene-specific mRNA analyses using the CTX96 Real-Time PCR System (Bio-Rad). Quantitative PCR amplification reactions contained the same amount of reverse transcription product (50 ng), including 7.5 μl of 2× iQSYBR Green Mix (Bio-Rad) and 100–300 nM forward and reverse primers, in a final volume of 15 μl. The primer sequences are listed below. Primer efficiency was obtained from the standard curve and integrated for the calculation of relative gene expression, which was based on real-time PCR threshold values of different transcripts.

mGpr37l1 forward: *GCATTGTGTGGCACAGCTAC*

mGpr37l1 reverse: *AAACTGGAAATGCCAGCGTG*

mKcnj10 forward: *TGCGGAAGAGTCTCCTCATTGG*

mKcnj10 reverse: *GTCTGAGGCTGTGTCTACTTGG*

mGapdh forward: *AGGTCGGTGTGAACGGATTTG*

mGapdh reverse: *GGGGTCGTTGATGGCAACA*

hGPR37L1 forward: *ACGAGATCACCAAGCAGAGGCT*

hGPR37L1 reverse: *GCACAGAGGCTGAAAGTCGTGA*

hKCNJ3 forward: *GATCTCCATGAGGGACGGAAAAC*

hKCNJ3 reverse: *GAAGGAACTCACCCTCAGGTGT*

hGAPDH forward: *GTCTCCTCTGACTTCAACAGCG*

hGAPDH reverse: *ACCACCCTGTTGCTGTAGCCAA*

Preparation of plasma membrane (PM) fraction

Plasma membrane (PM) fraction was prepared from mouse or human DRG tissues and HEK cells using Mem-PER Plus Kit (Thermo Scientific, Cat No. 89842) and manufacture recommend protocol after biotinylation/streptavidin-magnetic bead (Thermo Scientific, Cat No.88817) pull down. Tissues or cells were harvested and washed out using a cold cell washing buffer. The tissues/cells were lysed by cell permeabilized buffer containing protease inhibitor (Sigma, Cat No. P8380) and phosphatase inhibitor (Sigma, Cat No.524631) using mechanical dissociation and ultrasonication. The lysate was centrifugated and the pellet was resuspended with PER lysis buffer containing protease and phosphatase inhibitor cocktail (Sigma).

Western blot

Protein samples were prepared from transfected cells, lipid pull-down beads, and DRGs. Tissue and cells were placed on ice and lysed with ice-cold RIPA buffer (Sigma, Cat No. R0278) with Protease Inhibitor Cocktail Tablet (pH 7.4) (Roche Diagnostics, Cat No. 5892988001). The cell lysates were centrifugated to remove insoluble debris and the protein level was detected via BCA assay. The supernatant was mixed with 4x Laemmli buffer (BioRad, Cat No. 1610747) and boiled for 10 min (100 °C, containing BME for reducing Bis-Tris PAGE). Protein samples were electroporated on 4-20% gradient SurePAGE™ Bis-Tris gel (Genscript) or 4-20% Tris-glycine gel (BioRad) and blotted on a PVDF membrane (BioRad). Ponceau S staining was used for the detection of total proteins. The primary antibody was incubated with 1% BSA at 4℃ overnight. We used the following primary antibodies: anti-GPR37L1 antibody (Bioss, Rabbit, 1:1000, Cat No. bs-15390R), anti-Kir4.1 antibody (Thermo Scientific, rabbit, 1:1000, Cat No. 12503-1-AP), Kir3.1/KCNJ3 antibody (Santacruz, mouse, 1:500, Cat No. sc-365457), anti-TfR antibody (Santa Cruz, mouse, 1:500, Cat No.sc-32272), anti-flag antibody (Cell signaling, Rabbit, 1:1000, Cat No.14793), and anti-GAPDH antibody (Proteintech, mouse, 1:2000, Cat No. 60004). Blots were further incubated with an HRP-conjugated secondary antibody (Santacruz, mouse, 1:2000, Cat No. sc-2357) or m-IgG Fc BP-HRP (Santacruz, 1:5000, Cat No sc-525409) developed in ECL solution (Pierce). Some blots were also incubated with an Alexa Fluor® 647-conjugated donkey anti-rabbit IgG (Jackson ImmunoResearch, 1:1000, Cat No. 711-605-152) or with Alexa Fluor® 647-conjugated donkey anti-guinea pig (Jackson ImmunoResearch, 1:1000, Cat No. 706-605-148) and visualized in ChemiDoc (BioRad) using

chemiluminescence or Cy5 detection protocol. Protein signal intensity was quantified by Image Lab 6.1 (BioRad).

Immunohistochemistry

Mice were deeply anesthetized with isoflurane and perfused through the ascending aorta with PBS, followed by 4% PFA. After the perfusion, L4-L5 DRGs were removed from the mice and post-fixed in the same fixative overnight. Human DRGs were obtained from the National Disease Research Interphase (NDRI) and fixed in 4% PFA overnight. The samples were then dehydrated with a 10% to 30% sucrose gradient, embedded in Tissue-Tek O.C.T., and sliced into sections (14 μm) in a cryostat. The sections were blocked with 5% donkey serum and 0.2% triton X-100 for 1 h at room temperature, then incubated overnight at 4 °C with the following primary antibodies: anti-GPR37L1 antibody (Alomone Labs, Rabbit, 1:500, Cat No. AGR-050), anti-GS antibody (Novus Biologicals, Rabbit, 1:1000, Cat No. NBP2-32241), anti-FABP7 (Neuromics, Mouse,1:1000, Cat No.MO22188), and anti-KCNJ10/Kir4.1 antibody (Thermo Scientific, Guinea pig, 1:800, Cat No. PA5-111798). The sections were washed in PBS and incubated with the following secondary antibodies (1:500, Biotium) for 2 h at room temperature: CF633-donkey anti-rabbit (Cat No. 20125), CF568 donkey anti-guinea pig (Cat No. 20377), and CF488A donkey anti-mouse (Cat No. 20952). For clarity, channel colors were exchanged in the presented mouse DRG images without disrupting the signal using ImageJ. DAPI (1:1,000, Thermo Scientific, Cat No. 62248) was used to stain the cell nuclei in tissue sections. The sections were then washed with PBS, mounted in Fluoromount G mounting medium (Southern Biotech, Cat No. 0100-01), and observed under a confocal laser scanning microscope (SP5 Inverted confocal-LSRC, Leica Microsystems). Some stained sections were also examined with a Leica SP5 or Zeiss 880 confocal microscope with Z-stack and Tile Scan. The maximum projected and stitched images were produced using the Zeiss Zen software or with ImageJ.

Patch-clamp recordings in mouse SGCs in whole-mount DRG preparation

Under urethane anesthesia, mice were rapidly euthanized, followed by careful isolation of lumbar DRGs placed in the oxygenated artificial cerebral spinal fluid (ACSF). DRGs were briefly digested (20 min) using an enzymatic mixture consisting of collagenase A (1 mg/mL) and Trypsin (0.25% original solution). Intact DRGs were then incubated in ACSF oxygenated with 95% O_2 and 5% CO_2 at 34°C. Following incubation, DRGs were transferred to a recording chamber and continuously perfused (~3 ml/min) with ACSF. SGCs in whole mouse DRG could be visualized using a 40x water-immersion objective on an

Olympus BX51WI microscope. The round or fusiform-shaped cell bodies of SGCs have small sizes (<10 μm) but are visible near the edges of DRG neurons. Patch pipettes (Chase Scientific Glass Inc.) were pulled and filled with a pipette solution containing (in mM): 126 potassium gluconate, 10 NaCl, 1 $MgCl_2$, 10 EGTA, 2 Na-ATP, and 0.1 Mg-GTP, adjusted to pH 7.3 with KOH. The resistance of pipettes was 10-12 MΩ. A Whole-cell patch-clamp configuration was made on SGCs at room temperature using a Multiclamp 700B amplifier (Axon Instruments, Union City, CA). Under voltage clamp, at a holding potential of -80 mV, inward or outward currents were triggered by voltage steps beginning at -160 mV and increasing by 20 nM every 200 ms to a maximum of +40 mV (14). To isolate the inwardly rectifying potassium current (Kir), potassium currents from the same cells were recorded in the absence and presence of 100 μm extracellular barium for the blockade of the Kir4.1 channel (15, 16). The Kir4.1 currents were obtained by digitally subtracting those currents in the absence and presence of barium.

Ti^+ influx assay

Ti^+ flux assay was conducted using the manufacture's and standard protocol (17). For examining Ti^+ flux in GPR37L1, KCNJ3, KCNJ3/KCNJ10-expressing Hek293 cells, and WT/E296K-GPR37L1-expressing human SGCs, cells were dislodged from tissue culture flasks using TrypLE Express reagent (Gibco, Cat No. 12604013) and transferred to a 50 mL centrifuge tube, and centrifuged at 500g for 2 min. The supernatant solution was removed by aspiration, and the pellet was resuspended at a concentration of 1000 cells/μL in a cell culture medium. Then 100 μl/well of the cell suspension was transferred to 96-well plates. The seeded cells were incubated overnight in a humidified 5% CO2 cell culture incubator at 37 °C in low FBS media (2%). After overnight incubation, the cell culture medium was replaced with thallium influx dye-loading solution consisting of assay buffer (Hanks Balanced Salt Solution + 10 mM HEPES), 0.04% (w/v) Pluronic F-127 (Sigma), 1 μM of Thallos-AM (Ion bioscience) and 1 x TRS solution. Following a 60 min incubation at room temperature, the dye-loading solution was replaced with HBSS + 2 mM Ca^{2+} and + 2 mM Mg^{2+} 90 μl/well of assay buffer, and the plates were loaded into an Infiniti-M200 pro reader (Tecan, Männedorf, Switzerland). Data were acquired at (excitation 480, emission 520 nm) for 30 s intervals, followed by the addition of 10 μL/well of test compounds (30 min for KCNJ3 cell line or 1 h for KCNJ10 cell line). After baseline measurement for 5 min, 10 μl/well Ti^+ stimulation was injected (at a rate of 100 μl/sec), and fluorescence was measured for 10 min. The Tl^+ stimulus buffer consisted of 125 mM $KHCO_3$, 1.8 mM $CaSO_4$, 1 mM $MgSO_4$, 5 mM glucose, 5 mM Tl_2SO_4, 10 mM HEPES, pH 7.4. The cover-glassed cultures of mouse and human SGCs were imaged using an Orca6 camera (Hamamatsu)

every 3 sec with excitation at 480 nm and emission at 520 nm filters after thallium influx dye-loading solution. The imaging was performed using the MetaFluor software (Molecular Devices) in HBSS buffer supplemented with 2 mM Mg^{2+} and 2 mM Ca^{2+}. The activity was measured following stimulation with 500 µM Ti^{+} solution.

Genome-wide association

Genome-wide association tests were performed in the large UK Biobank project cohort comprised of half a million participants (41, 67). Information about chronic pain reports collected at the visit was available at eight body sites: head (headaches), face, neck/shoulder, stomach/abdominal, back, hip, knee, and widespread. Control subjects were those that answered, "none of the above" at field 6159 for the question "In the last month have you experienced any of the following that interfered with your usual activities?". Case subjects for chronic pain at body site X (among the eight listed) were those that answered "yes" to the question "Have you had pains for more than 3 months?"; fields are 3571 (back), 4067 (face), 2956 (widespread), 3799 (headaches), 3414 (hip), 3773 (knee), 3404 (neck/shoulder), and 3741 (stomach/abdominal). Input genotyping data for association tests were from whole-exome sequencing (in VCF format), available for 200K individuals. Variants were annotated to GRCh38 reference provided by UK Biobank (http://biobank.ndph.ox.ac.uk/ukb/refer.cgi?id=3803) with Ensembl Variant Effect Predictor (VEP, release 99) (68). Rare coding variants were tested, and we required a minor allelic frequency of less than 0.01 and a minor allele count greater than 5. We used SAIGE (version 0.44.2) (69) to perform the tests, as it considers cryptic relatedness and guards against false-positive associations in the face of case-to-control imbalance (due to the cross-sectional nature of the cohort) by means of the saddle point approximation method (70). Covariables were: age, age squared, sex, genotyping platform, recruitment centers, and the first 40 principal genetic components. Subjects were of White British origin (field 22006). Discarded subjects were those that opted out of the study, failed genotyping or imputation quality controls, or displayed sex aneuploidy or mismatched declared / genetically-determined sex. Retained SNPs displayed at least five counts of the minor allele in selected cases or control subjects. The meta-analysis at a given variant position across all eight chronic pain sites was performed using the inverse variance-based weighting scheme (71). A combined Annotation Dependent Depletion score (CADD v1.6) (43) was used to estimate the deleteriousness of the variants. It computed PHRED-scale CADD scores for variants, which provided a relative order compared to all existing variants in the human genome. Higher CADD scores for higher risk of deleteriousness.

Reference List

1. Han Q, Liu D, Convertino M, Wang Z, Jiang C, Kim YH, et al. miRNA-711 Binds and Activates TRPA1 Extracellularly to Evoke Acute and Chronic Pruritus. *Neuron.* 2018;99(3):449-63 e6.
2. Luo X, Huh Y, Bang S, He Q, Zhang L, Matsuda M, and Ji RR. Macrophage Toll-like Receptor 9 Contributes to Chemotherapy-Induced Neuropathic Pain in Male Mice. *J Neurosci.* 2019;39(35):6848-64.
3. Brigham NC, Nofsinger R, Luo X, Dreger NZ, Abel AK, Gustafson TP, et al. Controlled release of etoricoxib from poly(ester urea) films for post-operative pain management. *J Control Release.* 2021;329:316-27.
4. Hylden JL, and Wilcox GL. Intrathecal morphine in mice: a new technique. *EurJPharmacol.* 1980;67(2-3):313-6.
5. Parisien M, Khoury S, Chabot-Dore AJ, Sotocinal SG, Slade GD, Smith SB, et al. Effect of human genetic variability on gene expression in dorsal root ganglia and association with pain phenotypes. *Cell Rep.* 2017;19(9):1940-52.
6. Bang S, Xie YK, Zhang ZJ, Wang Z, Xu ZZ, and Ji RR. GPR37 regulates macrophage phagocytosis and resolution of inflammatory pain. *J Clin Invest.* 2018;128(8):3568-82.
7. Poulsen JN, Larsen F, Duroux M, and Gazerani P. Primary culture of trigeminal satellite glial cells: a cell-based platform to study morphology and function of peripheral glia. *Int J Physiol Pathophysiol Pharmacol.* 2014;6(1):1-12.
8. Esguerra M, Siretskiy A, Bello X, Sallander J, and Gutierrez-de-Teran H. GPCR-ModSim: A comprehensive web based solution for modeling G-protein coupled receptors. *Nucleic Acids Res.* 2016;44(W1):W455-62.
9. Morris GM, Huey R, Lindstrom W, Sanner MF, Belew RK, Goodsell DS, and Olson AJ. AutoDock4 and AutoDockTools4: Automated docking with selective receptor flexibility. *J Comput Chem.* 2009;30(16):2785-91.
10. Bowers KJ, Chow E, Xu H, Dror RO, Eastwood MP, Gregersen BA, et al. *Proceedings of the 2006 ACM/IEEE conference on Supercomputing*. Tampa, Florida: Association for Computing Machinery; 2006:84–es.
11. Xu J, Wu S, Wang J, Wang J, Yan Y, Zhu M, et al. Oxidative stress induced by NOX2 contributes to neuropathic pain via plasma membrane translocation of PKCepsilon in rat dorsal root ganglion neurons. *J Neuroinflammation.* 2021;18(1):106.
12. Pucci F, Schwersensky M, and Rooman M. Artificial intelligence challenges for predicting the impact of mutations on protein stability. *Curr Opin Struct Biol.* 2022;72:161-8.
13. Pancotti C, Benevenuta S, Birolo G, Alberini V, Repetto V, Sanavia T, et al. Predicting protein stability changes upon single-point mutation: a thorough comparison of the available tools on a new dataset. *Brief Bioinform.* 2022;23(2).
14. Zhang H, Mei X, Zhang P, Ma C, White FA, Donnelly DF, and Lamotte RH. Altered functional properties of satellite glial cells in compressed spinal ganglia. *Glia.* 2009;57(15):1588-99.
15. Doupnik CA, Davidson N, and Lester HA. The inward rectifier potassium channel family. *Curr Opin Neurobiol.* 1995;5(3):268-77.
16. Tong X, Ao Y, Faas GC, Nwaobi SE, Xu J, Haustein MD, et al. Astrocyte Kir4.1 ion channel deficits contribute to neuronal dysfunction in Huntington's disease model mice. *Nature neuroscience.* 2014;17(5):694-703.
17. Weaver CD. Thallium Flux Assay for Measuring the Activity of Monovalent Cation Channels and Transporters. *Methods Mol Biol.* 2018;1684:105-14.
18. Avraham O, Deng PY, Jones S, Kuruvilla R, Semenkovich CF, Klyachko VA, and Cavalli V. Satellite glial cells promote regenerative growth in sensory neurons. *Nat Commun.* 2020;11(1):4891.
19. Yang L, Xu M, Bhuiyan SA, Li J, Zhao J, Cohrs RJ, et al. Human and mouse trigeminal ganglia cell atlas implicates multiple cell types in migraine. *Neuron.* 2022;110(11):1806-21 e8.

Supplemental Figures

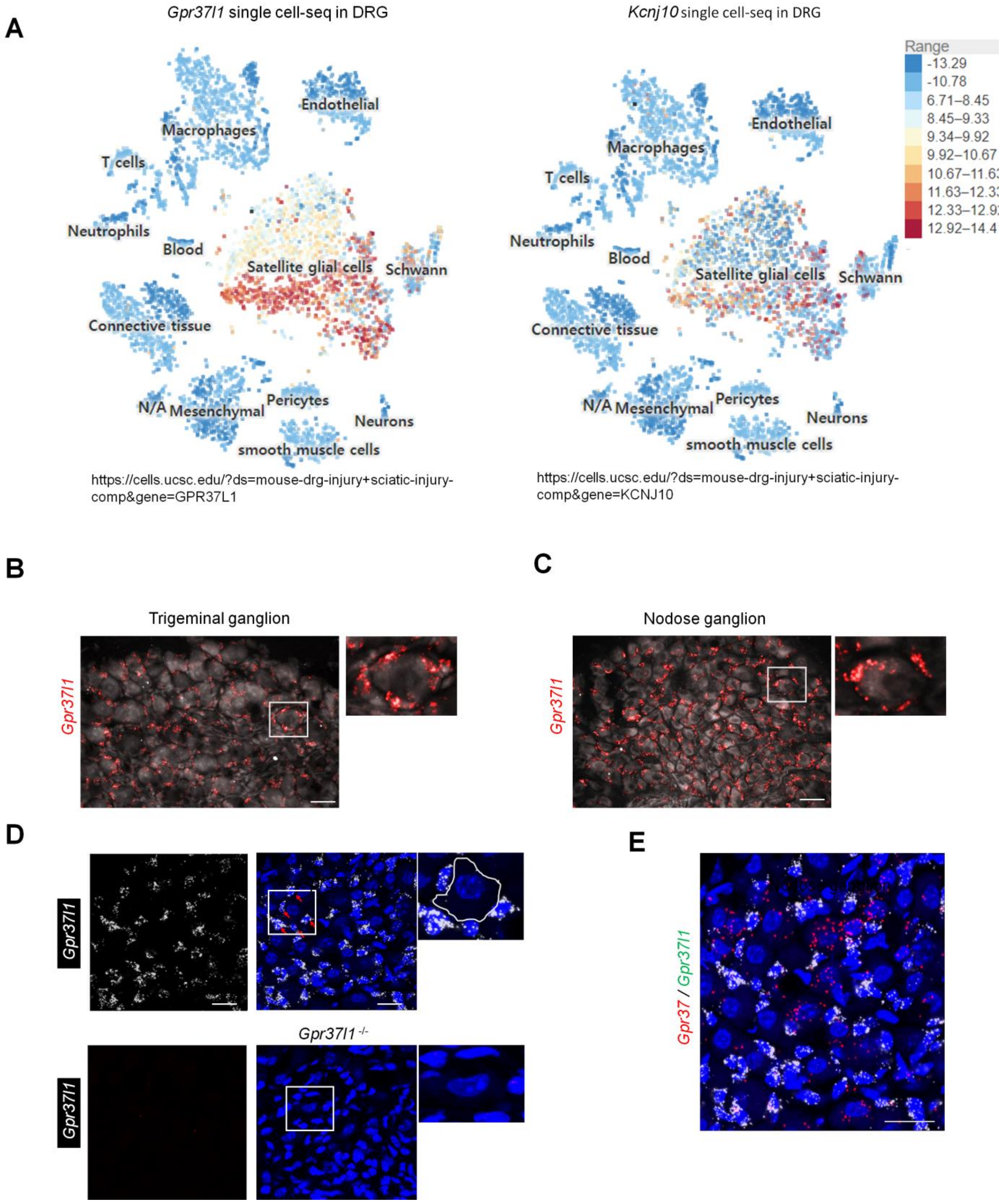

Supplemental Figure 1. Mouse RNA sequencing and human microarray analysis. (**A**) Single-cell RNAseq showing expression of *Gpr37l1* and *Kcnj10* transcripts in SGCs of mouse DRG tissues (18). (**B, C**) Images of RNAscope in situ hybridization (ISH) of *Gpr37l1* expression in trigeminal ganglion (TG, B) and nodose ganglion (NG, C). Small boxes are enlarged in the right panels. Neurons are lightly labeled with Nissl staining (white). Scales, 25 µm. (**D**) RNAscope images of *Gpr37l1* expression in NG of *Gpr37l1*$^{+/+}$ mice (WT, top) and *Gpr37l1*$^{-/-}$ mice (bottom). Small boxes are enlarged in the right panels. Scales, 25 µm. (**E**) Double staining of RNAscope ISH showing distinct expression of *Gpr37* (red) and *Gpr37l1* (white) in mouse NG. Scale = 25 µm.

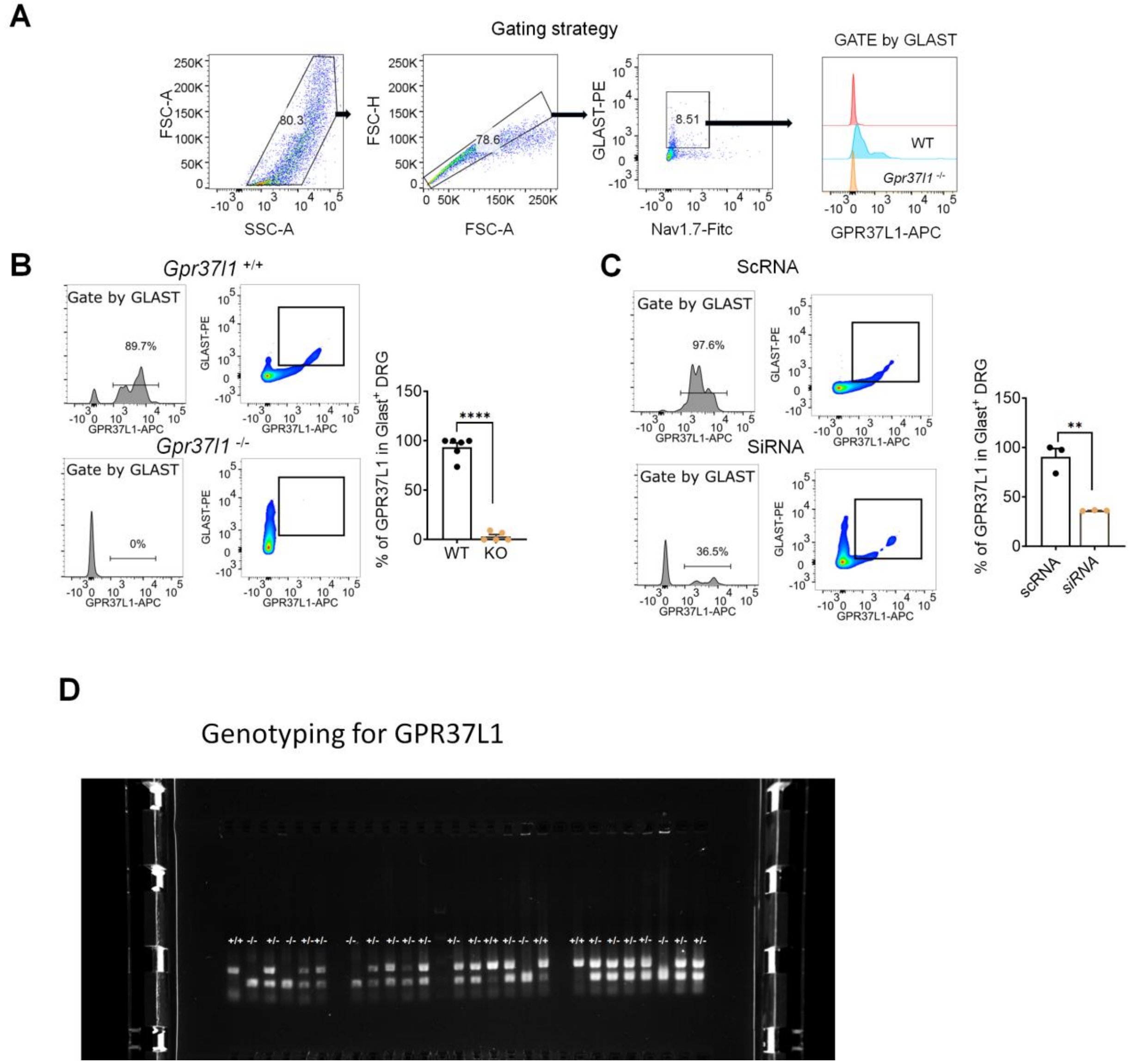

Supplemental Figure 2. Characterization of GPR37 expression in mouse DRG tissue by flow cytometry. (**A-C**) Flow cytometry data showing GPR37L1 expression in DRGs of *WT* and *Gpr37l1*$^{-/-}$ mice. (**A**) Gating strategy. **(B)** GPR37L1 expression in DRGs of *WT* and *Gpr37l1*$^{-/-}$ mice. Left, representative images of flow cytometry and histograms after gating by Glast-PE. Right, Quantification of GPR37L1+ cells in the Glast-PE+ population (*n* = 6 for WT and *n* = 5 for KO mice). **(C)** Left, representative images of flow cytometry and histogram after gating by Glast-PE in DRGs of scRNA or siRNA treated mice (related to Figure 3G). Right, Quantification of GPR37L1+ cells in the Glast-PE+ population (*n* = 3 mice/group). GLAST was used as a marker to isolate SGCs in DRGs. (**D**) Genotyping for *Gpr37l1* WT and mutant mice. Data are expressed as mean ± s.e.m. ****P<0.0001, **P<0.01, unpaired t-test.

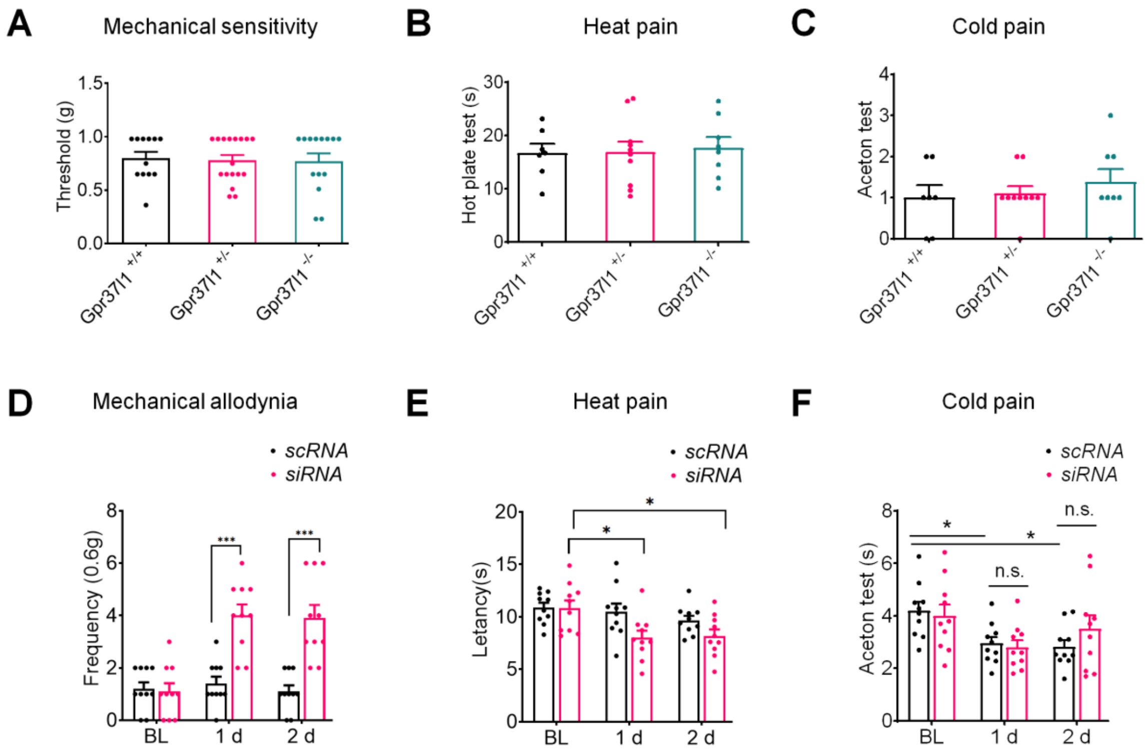

Supplemental Figure 3. Characterization of mechanical and thermal pain in WT mice and *Gpr37l1* mutant mice. (**A-C**) Baseline pain sensitivity in *Gpr37l1*$^{+/+}$ mice (*n = 12*), *Gpr37l1*$^{+/-}$ mice (*n* = 12)*, and Gpr37l1*$^{-/-}$ mice (*n* = 17), assessed in von Frey test (mechanical sensitivity, A), hot-plate test (heat sensitivity, B), and acetone test (cold sensitivity, C). (**D-F**) Mechanical and thermal hyperalgesia in mice treated with I.G. injection of *Gpr37l1* siRNA (*n* = 10) and control scRNA (*n* = 10). (**D**) Paw withdrawal frequency (0.6 g) showing the siRNA-induced mechanical allodynia at 1d and 2d. (**E**) Hargreaves test showing siRNA-induced heat hyperalgesia at 1d. (**F**) Acetone test showing cold sensitivity following the siRNA treatment. Data are expressed as mean ± SEM and statistically analyzed by One-Way ANOVA with Turkey's post-hoc test (A-C) or Two-Way ANOVA with Bonferroni's post-hoc test (scRNA vs siRNA, D-F). *P<0.05, ***P<0.001; n.s., not significant.

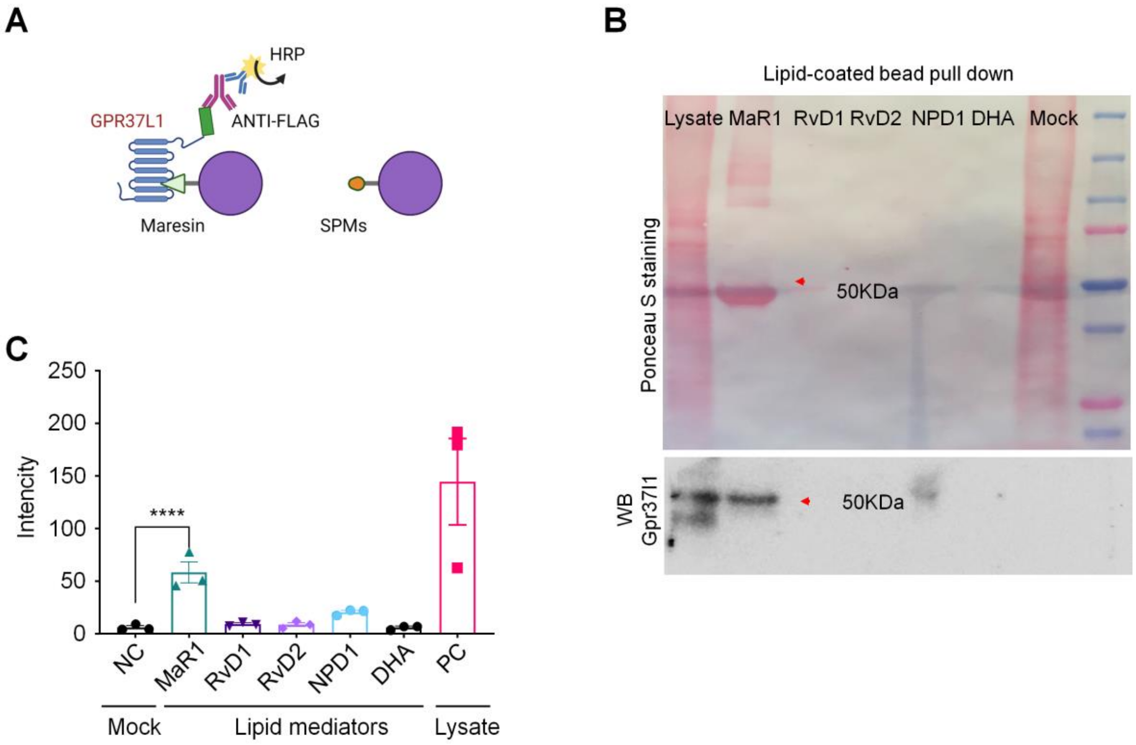

Supplemental Figure 4. GPR37L1 binds with MaR1. Lipid pull-down assay showing MaR1 binding to GPR37L1 in GPR37L1-expressing HEK293 cells. **(A)** Schematic of the lipid pull-down assay using agarose bead-coated SPM (MaR1). **(B)** Top, Ponceau S staining; red arrowhead indicates the specific band at 50 kDa. Bottom: Anti-FLAG Western blot showing the GPR37L1 band, indicated by red arrowhead. One microgram of protein was loaded. (**C**) Quantification of the intensity of immune blots; DRG lysate and Mock were used as positive control (PC) and negative control (NC). *n* = 3 repeats. Data are expressed as mean ± s.e.m. and analyzed by One-Way ANOVA followed by Tukey's post-hoc test (C). ****P<0.0001.

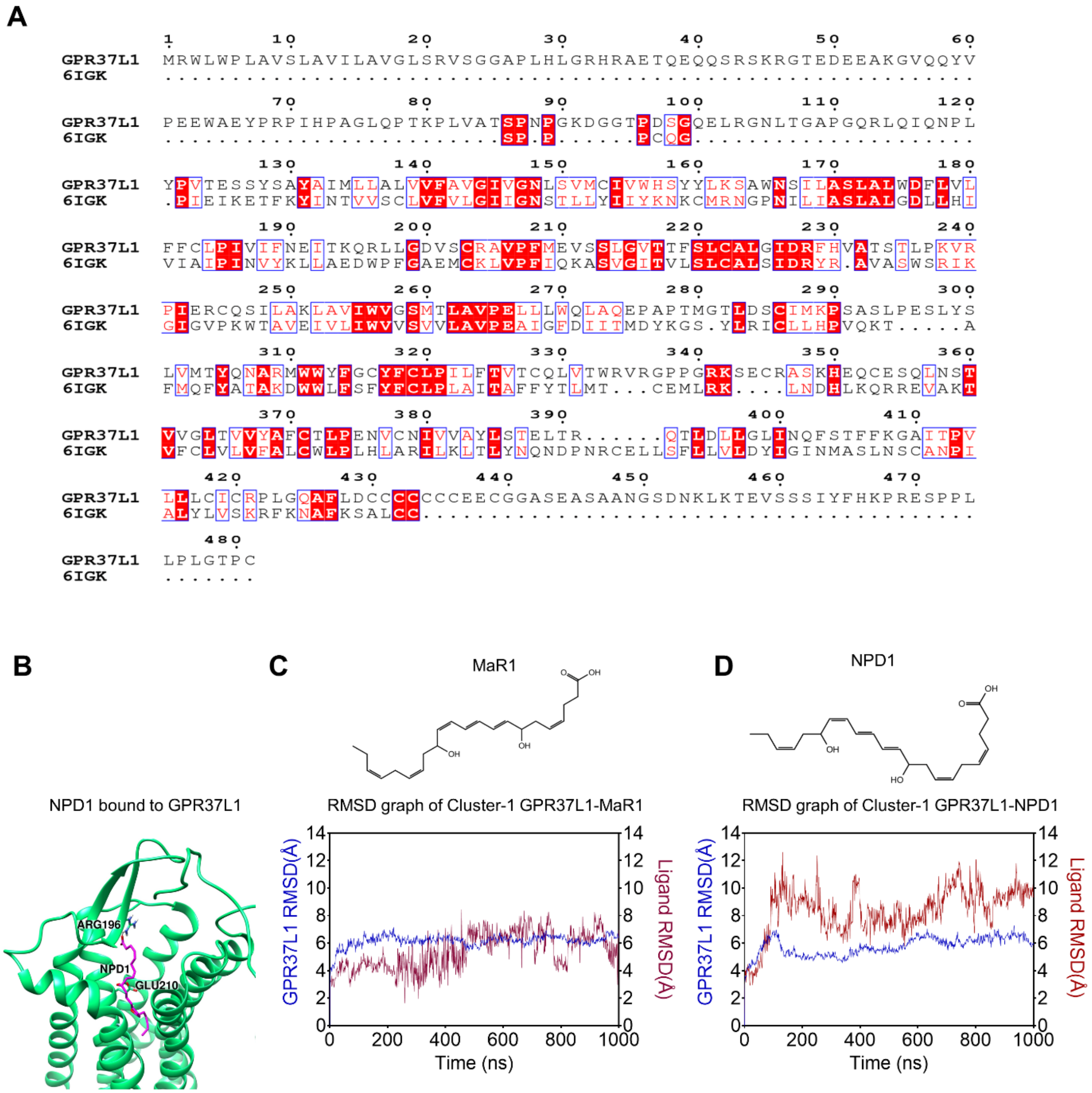

Supplemental Figure 5. Computer simulation for the interaction of GPR37L1 and MaR1. (**A**) Alignment of amino-acid sequences of a/the crystallized construct of human EDNRB (PDB: 6IGK) and human GPR37L1 (UniProt ID: O60883). Conservation of the residues is indicated as follows: red panels for completely conserved; red letters for partly conserved; and black letters for not conserved. Note there is a 30.23% homology between these two genes. **(B)** The overall structure of hGPR37L1 (Green) complex with NPD1 (Magenta). **(C)** 1000 ns molecular dynamics simulation of the GPR37L1-MaR1 complex (red) or GPR37L1(blue). **(D)** 1000 ns molecular dynamics simulation of the GPR37L1-NPD1 complex (red) or GPR37L1(blue). Note that MaR1, but not NPD1, has stable interaction with GPR37L1.

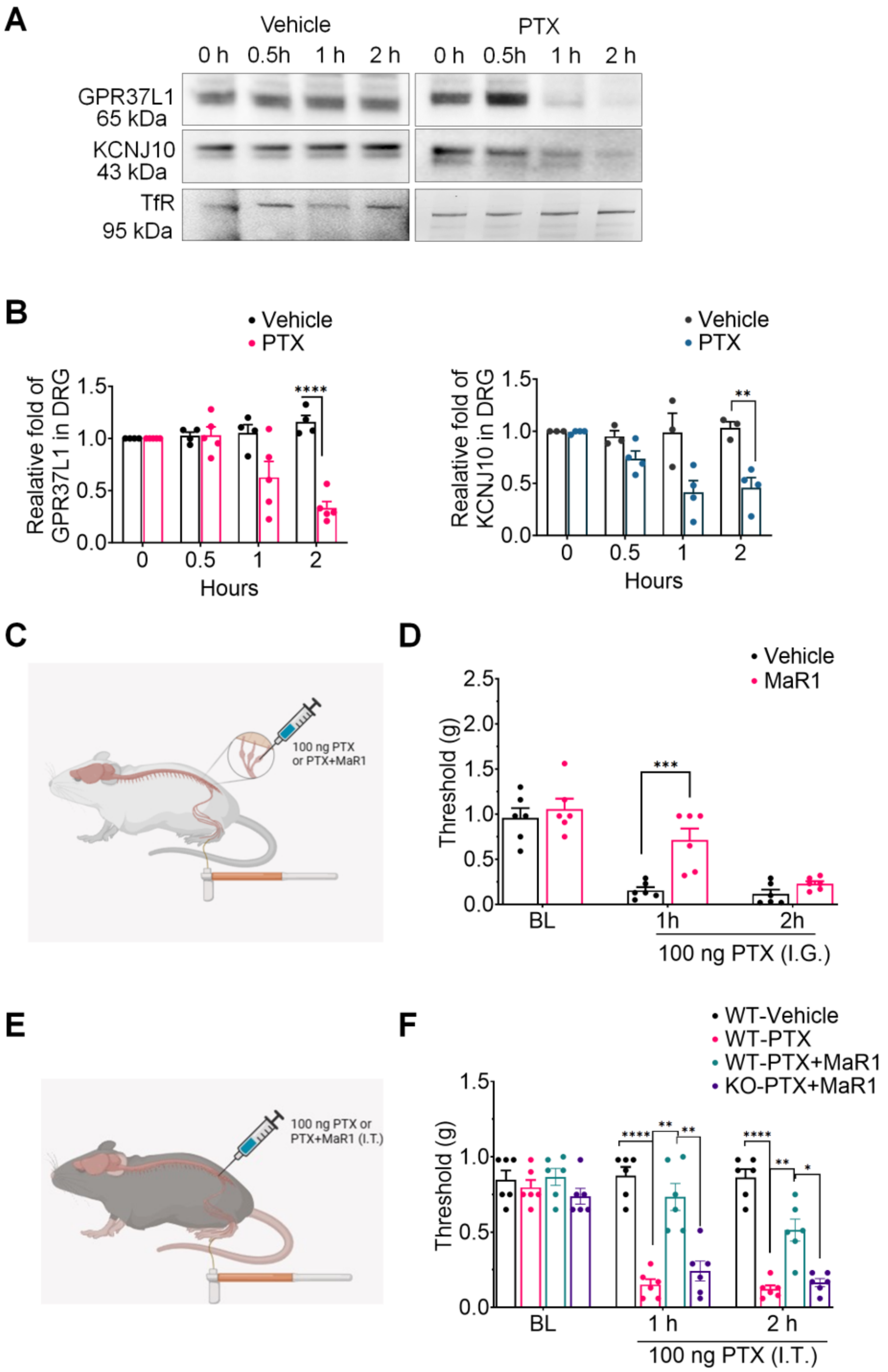

Supplemental Figure 6. Chemotherapy causes rapid downregulations of plasma membrane (PM) expression of GPR37L1 and KCNJ10 in whole mount DRGs. (A) Time course of PM expression of GPR37L1 and KCNJ10 following treatment of vehicle (left, three parallel gels) and paclitaxel (PTX, 1 μM, right, three parallel gels). **(B)** Quantification of PM fraction of GRP37L1 (left) and KCNJ10 (right) expression. n = 3 for vehicle and n = 4 for PTX. **(C-D)** Unilateral intraganglionic (I.G.) microinjection of PTX or PTX+MaR1 induces acute mechanical allodynia in naïve animals. 100 ng MaR1 application

prevented PTX(100 ng I.G. injection)-induced acute mechanical allodynia (n = 6). **(E-F)** Intrathecal (I.T.) injection of PTX or PTX+MaR1 in *Gpr37l1*$^{+/+}$ or *Gpr37l1*$^{-/-}$ mice. 100 ng MaR1 attenuated the PTX induced acute mechanical allodynia in *Gpr37l1*$^{+/+}$ mice not *Gpr37l1*$^{-/-}$ mice (n = 6). Data are expressed as mean ± s.e.m. and analyzed by Two-Way ANOVA followed by Tukey's post-hoc test (B) and Bonferroni's post-hoc test (D), and Two-Way RM ANOVA followed by Tukey's post-hoc test (F). *P<0.05, **P<0.01, ***P<0.001, ****P<0.0001.

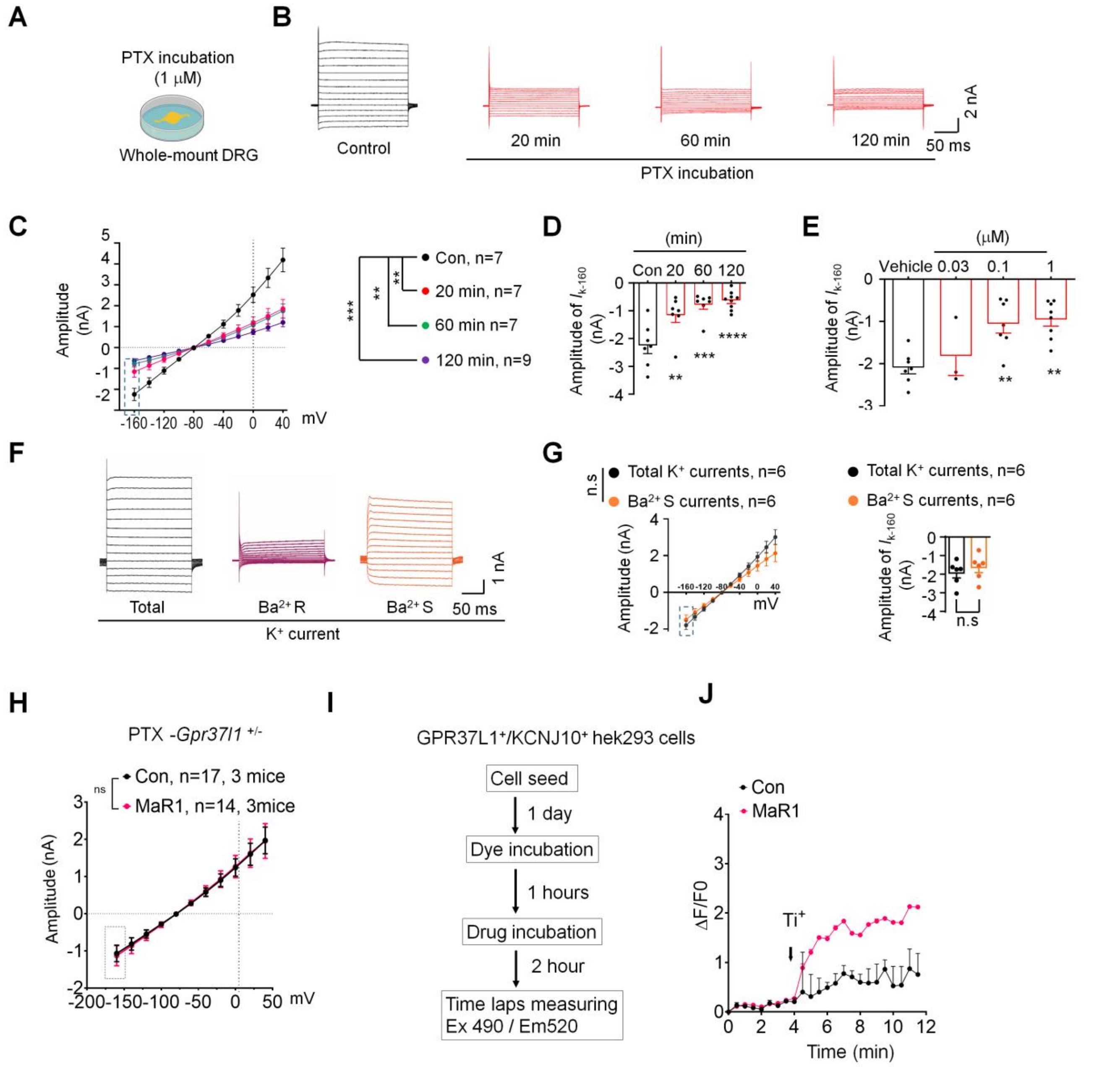

Supplemental Figure 7. Characterization of K^+ currents in SGCs of whole mount DRGs and Thallium (Ti^+) influx assay in HEK293 cells. (A) Schematic diagram for whole-mount SGC recording. **(B)** K^+ current traces in SGCs at different times after PTX treatment (1 μM, 10 min 20 min, 60 min, and 120 min) were recorded using the step voltage injection protocol. **(C)** Voltage-current curves in SGCs at different times after PTX treatment. **(D)** Quantification of the amplitude of -160 mV currents in (B) and (C). **(E)** Quantification of the amplitude of -160 mV currents at different doses of PTX (vehicle, 0.03 *n* = 3, 0.1,1 μM, *n* = 7 cells) at 20 min. **(F-G)** Isolation of Barium sensitive currents in SGCs in whole-mount

DRGs. **(F)** Example traces of Barium sensitive currents in SGCs. **(G)** Left, average voltage-current curves in SGCs for Barium sensitive or insensitive currents (*n* = 6 cells, left). Right, quantification of -160 mV currents for Barium sensitive and total currents (*n* = 6 cells, right). (**H**) Average I/V curves in *Gpr37l1*$^{+/-}$ SGCs after treatment of PTX (*n* = 17 cells) or PTX + MaR1 (*n* = 14 cells). **(I-J)** 96well plate Ti^{+} influx assay in GPR37L1 and KCNJ10 expressed HEK293 cells after MaR1 incubation. **(I)** Flowchart of Ti^{+} assay. **(J)** Representative traces of Ti^{+} influx in cells treated with 100 nM MaR1 (red, *n* = 3) or control vehicle (Con, black, *n* = 3) for 1h incubation. Data are expressed as mean ± SEM and analyzed by Two-way ANOVA with Tukey's post-hoc test (C, G left, H), One-way ANOVA with Tukey's post-hoc (D, E), and unpaired t-test (G right). $^{**}P<0.01$, $^{***}P<0.001$, $^{****}P< 0.0001$; n.s., not significant.

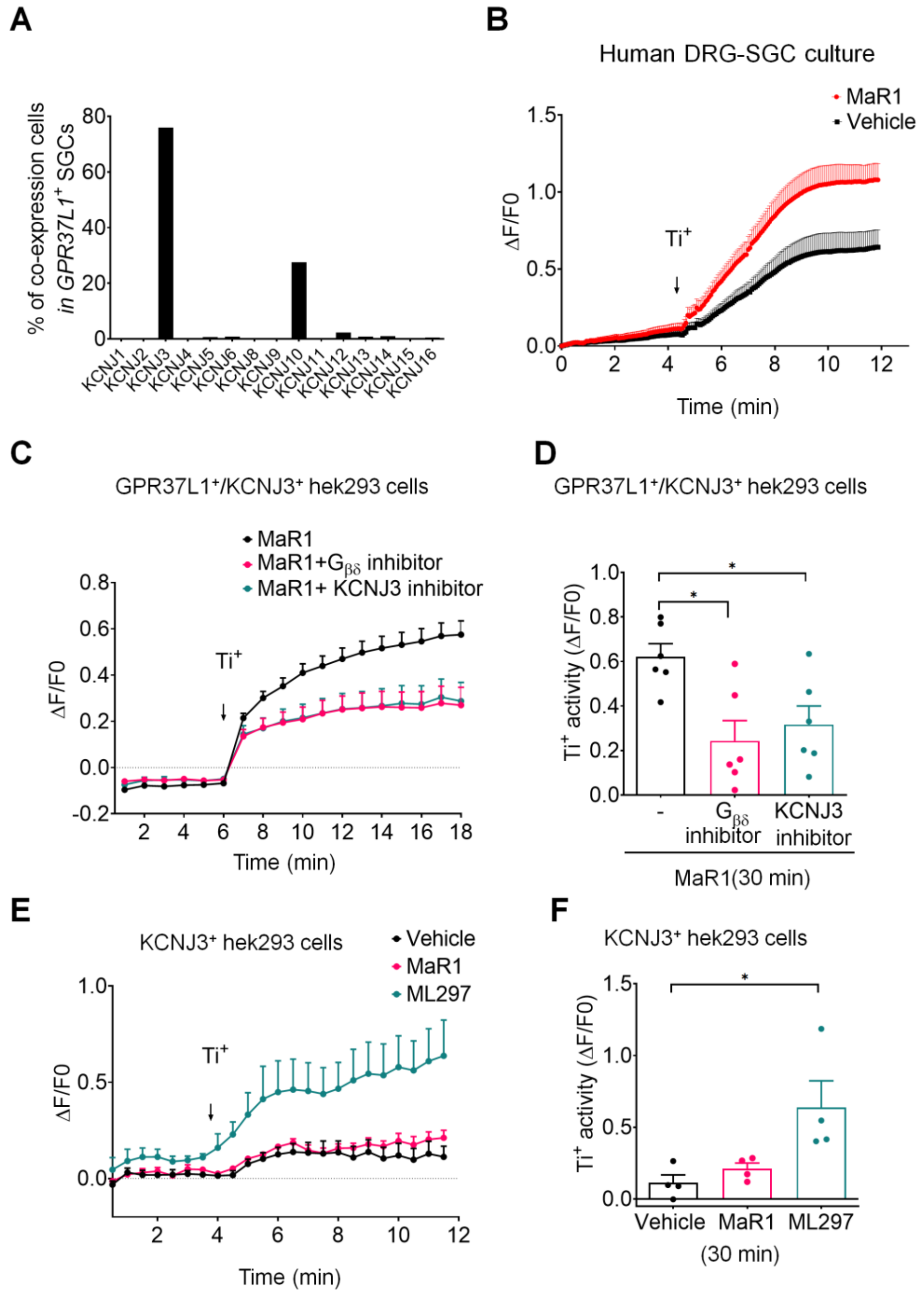

Supplemental Figure 8. GPR37L1 regulates KCNJ3-mediated K^+ influx in human DRG and HEK293 cells. (A) Co-expression percentage of *KCNJ3* and *KCNJ10* in GPR37L1+ SGCs from a human TG database of RNAseq (19). **(B)** Representative traces for Ti^+ influx assay of cultured human SGCs treated with MaR1 (100 nM, *n* = 7) or vehicle (*n* = 8). **(C-D)** Ti^+ influx assay in GPR37L1 and KCNJ3 expressing HEK293 cells. MaR1 increased Ti^+ influx, which was reduced by Gβγ inhibitor (Gallein, 1 µM) and KCNJ3 inhibitor (SCH 23390, 1 µM). The baseline value of the Ti^+ influx of the vehicle group

was subtracted. **(C)** Traces of time-dependent Ti^+ influx and the effects of Gβγ or KCNJ3 inhibitors (*n* = 14 at 30 min of drug incubation). **(D)** Quantification of Ti^+ influx at 10 min *(n* = 6 cultures). **(E-F)** Ti^+ influx assay in KCNJ3 expressing HEK293 cells. MaR1 and ML297 (KCNJ3 agonist) increased Ti^+ influx. The baseline value of Ti^+ influx of the vehicle group was subtracted. **(E)** Traces of time-dependent Ti^+ influx and the effects of (100 nM) KCNJ3 agonist, vehicle, and (30 nM) MaR1 (*n* = 4 cultures at 30 min of drug incubation). **(F)** Quantification of Ti^+ influx at 10 min *(n* = 4 cultures). Data are expressed as mean ± SEM and analyzed by One-way ANOVA with Tukey's post-hoc (D, F), *$P<0.05$.

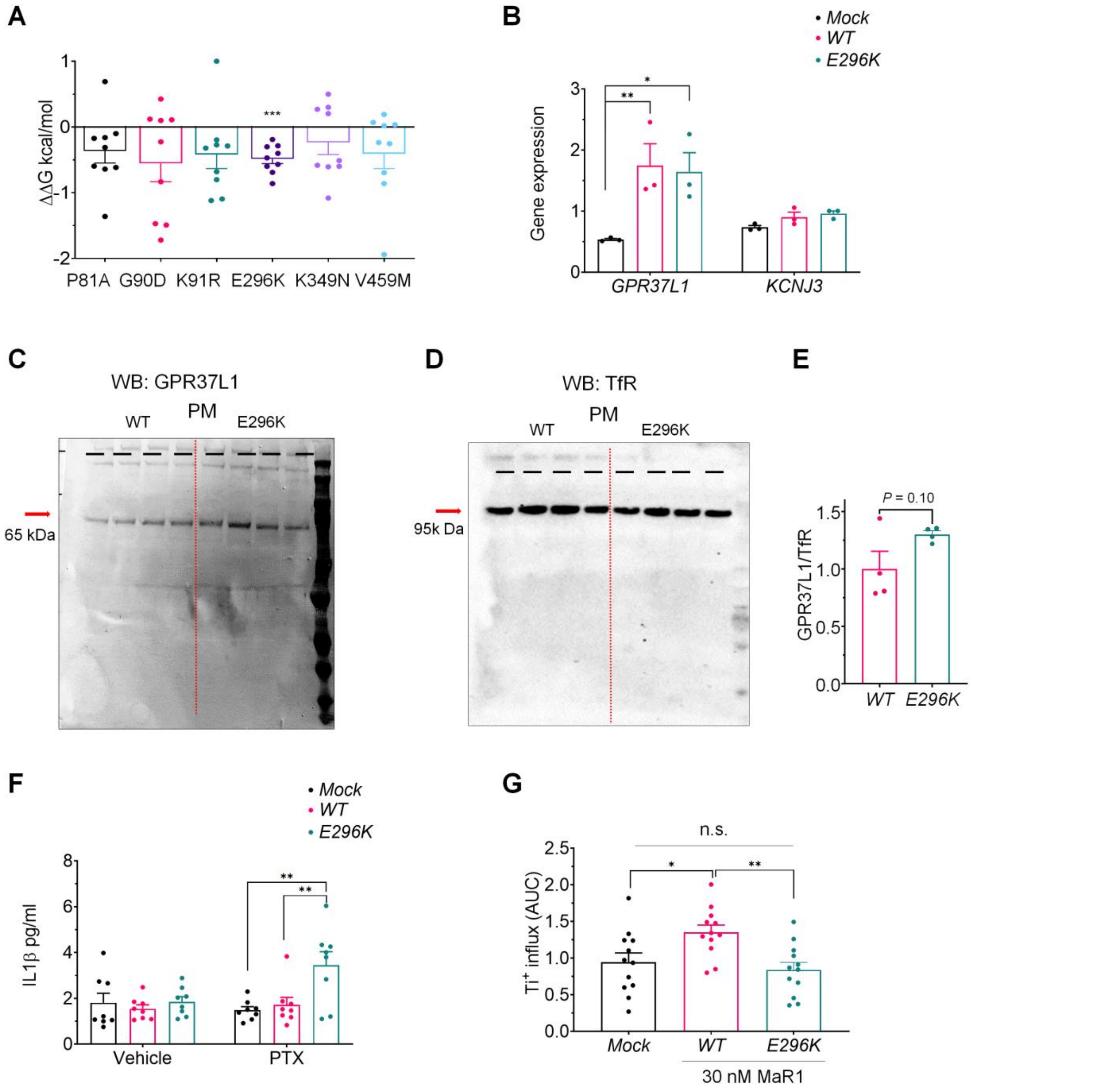

Supplemental Figure 9. Characterization of *GPR37L1* mutant in human SGCs and HEK293 cells. (A) Protein stability prediction using 9 different algorithms. GPR37L1 stability changes in different *GPR37L1* mutations were tested. ***P<0.001, one sample t-test. **(B)** Real-time PCR for the expression levels of *GPR37L1* and *KCNJ3* in mock, WT, or *E296K* mutant transfected human SGCs (n = 3). *P<0.05, **P<0.01, Two-Way ANOVA with Tukey's post-hoc test. **(C-E)** Plasma membrane expression levels of GPR37L1 (C) in WT and E296K mutant transiently transected HEK293 cells. TfR expression (parallel gel) was used as loading control for plasma membrane proteins (D). (E) Quantification of GPR37L1 band

intensity normalized to TfR. *n* = 4, *P* = 0.10, unpaired t-test. **(F)** ELISA analysis showing IL-1β production level in human SGCs after transfection of WT or mutant GPR37L1. PTX treatment (100 nM, overnight) increased IL-1β levels only in mutant-expressed human SGCs. *n* = 8, $**P<0.01$, Two-Way ANOVA with Tukey's post-hoc test. **(G)** Ti^+ influx activity in human SGCs showed that 30 nM MaR1 treatment significantly increased K^+ channel activity in WT but not E296K expressed cells. *n* = 12, $*P<0.05$, $**P<0.01$, One-Way ANOVA with Tukey's post-hoc test; n.s., not significant. All data are expressed as mean ± SEM,

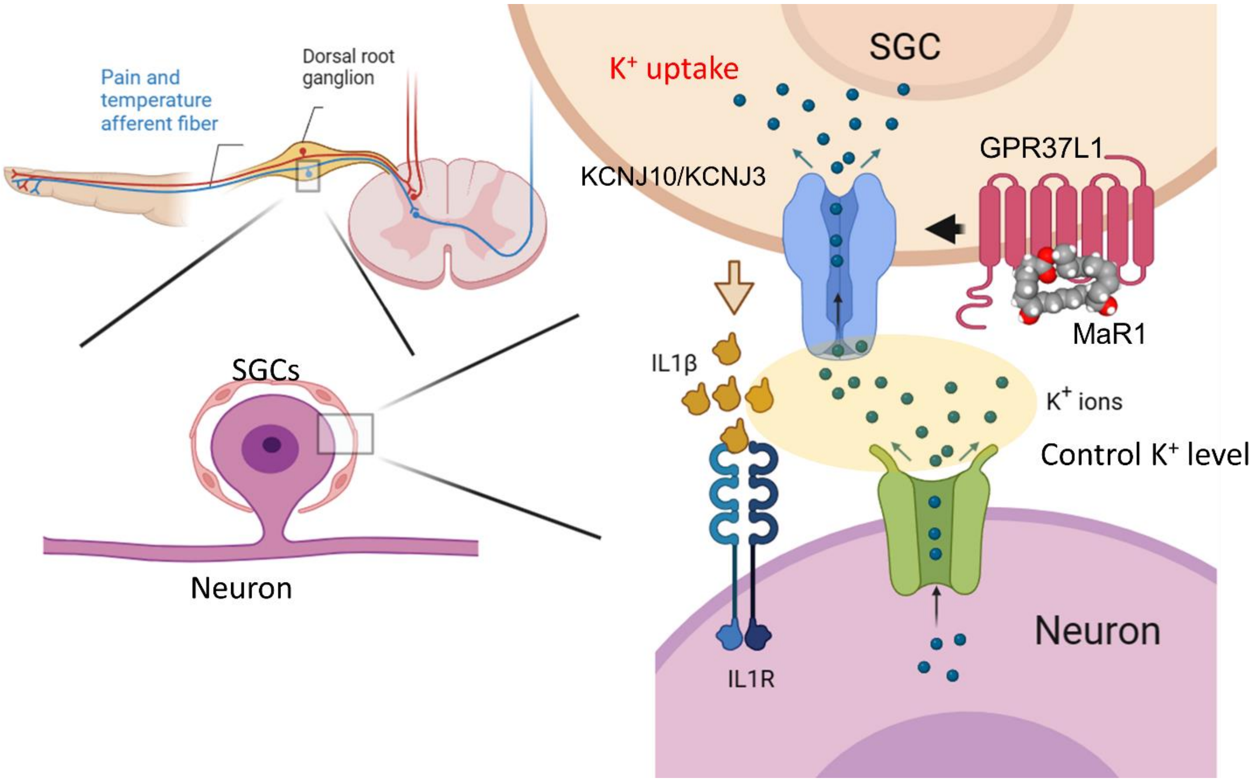

Supplemental Figure 10. Schematic illustration of GPR37L1-mediated regulation of K^+ channels in SGCs of DRG. Top left, pain transduction and transmission in peripheral and central axons of DRG neurons. Bottom left, SGCs surrounding a DRG neuron. Right, control of potassium channels signaling by GPR37L1 in SGCs. Neuronal excitation causes K^+ efflux, and extra K^+ can be uptaken by KCNJ10 in mouse SGCs and KCNJ3/KCNJ10 in human SGCs. Loss of K^+ uptake activity after chemotherapy and diabetes, as a result of downregulations of GPR37L1/KCNJ3/KCNJ10, will lead to neuropathic pain (CIPN and DPN). MaR1 treatment can activate GPR37L1, increase the K^+ uptake activity in SGCs, and regulate IL-1β production leading to a relief of neuropathic pain.

Supplemental Tables List

Supplemental Table 1: Total GPCR transcripts (A) and orphan GPCR transcripts (B) in mouse DRGs.

Supplemental Table 2: Total GPCR transcripts (A) and orphan GPCR transcripts (B) in human DRGs.

Supplemental Table 3: (A) SAIGE association tests for rare coding variants and a report of chronic pain at eight body sites. Columns are: “PainSite” for body site of chronic pain; “markerID” for variant ID; “Chr” for chromosome number of variant; “Pos” for GRCh38 genomic coordinate of variant; “Allele1” for the reference allele of variant; “Allele2” for the effective allele of variant; “rsid” for known rsid of variant; “AC2” for allele account of the effective allele; “AF2” for allele frequency of the effective allele; “AF2.Cases” and “AF2.Ctrls” for allele frequency of the effective allele in group cases and controls, respectively; “N.Cases”, “N.Ctrls”, and “N” for number of samples in group cases, controls, and total samples; “Consequence” for functional consequence predicted by VEP; “BETA” for effect size of effective allele; “SE” for standard error of “BETA”; “odds[95%]” for odds ratio and 95% confidence interval; “P” for raw P value; (B) Meta-analysis on SNP rs148475636 and rs767987863. Columns represent the same meaning as in Table 3A. Meta results are in row “Meta”.

Supplemental Table 4: Numbers and sexes of animals used in this study.

Supplemental Table 5: Human DRG donners and sexes used in this study. (A) Human DRGs from NDRI. (B) Information of human DRG donors with neuropathic pain (n = 3) and without neuropathic pain controls (n = 3).
