# Supplementary material for: Satellite glial GPR37L1 and its ligand maresin 1 regulate potassium channel signaling and pain homeostasis
Source: J Clin Invest. 2024 Mar 26;134(9):e173537. doi: 10.1172/JCI173537 (PMC11060744; doi:10.1172/JCI173537)
Supplement: Unedited blot and gel images [file jci-134-173537-s202.pdf]

Un cut gels

Figure 2C

Double staining
1st ab: GPR37L1, GAPDH
2nd ab: Rb-Cy3, Ms-Cy5

+/+: *Gpr37l1*$^{+/+}$
+/-: *Gpr37l1*$^{+/-}$
-/-: *Gpr37l1*$^{-/-}$

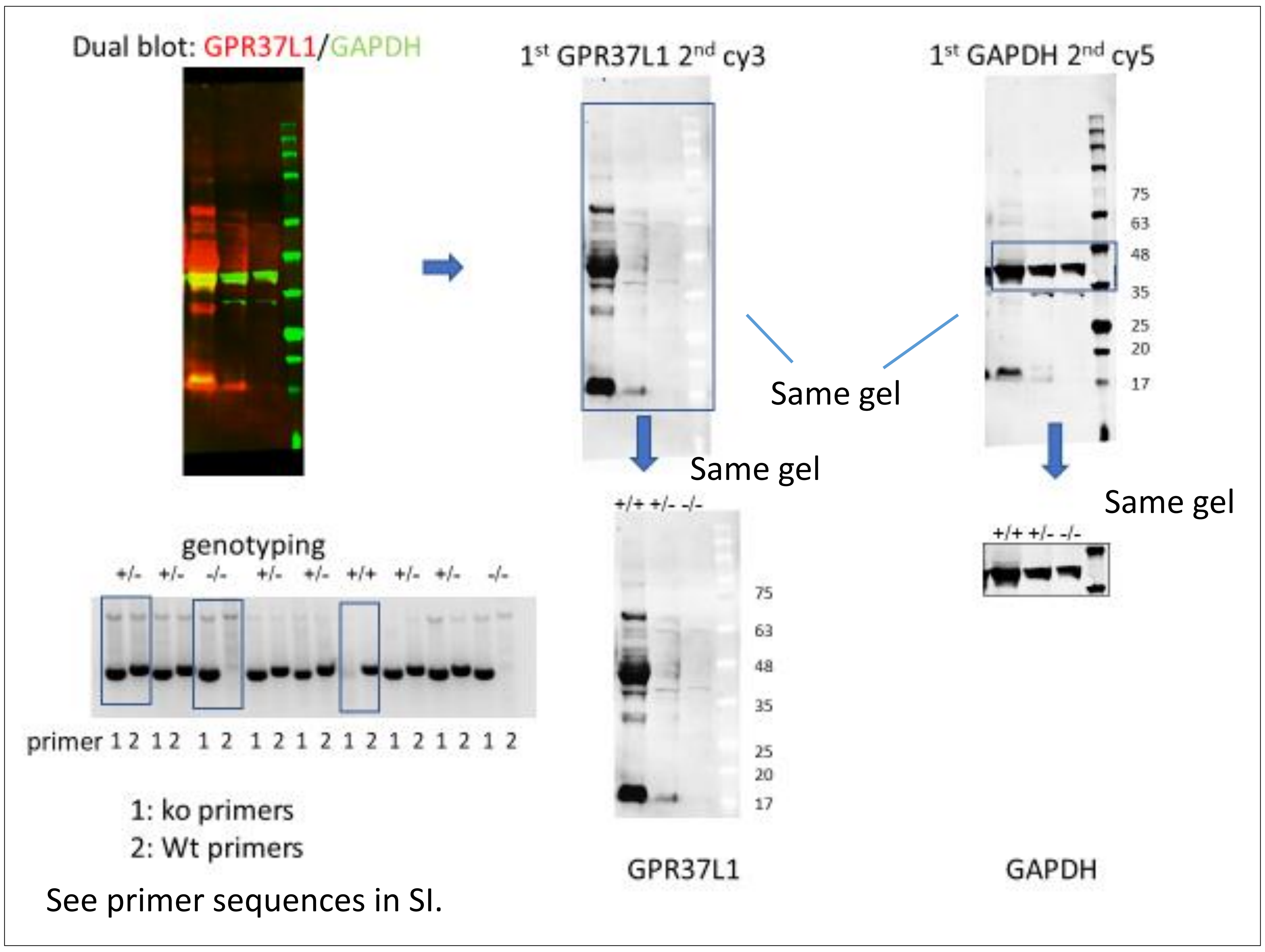

Figure 2F

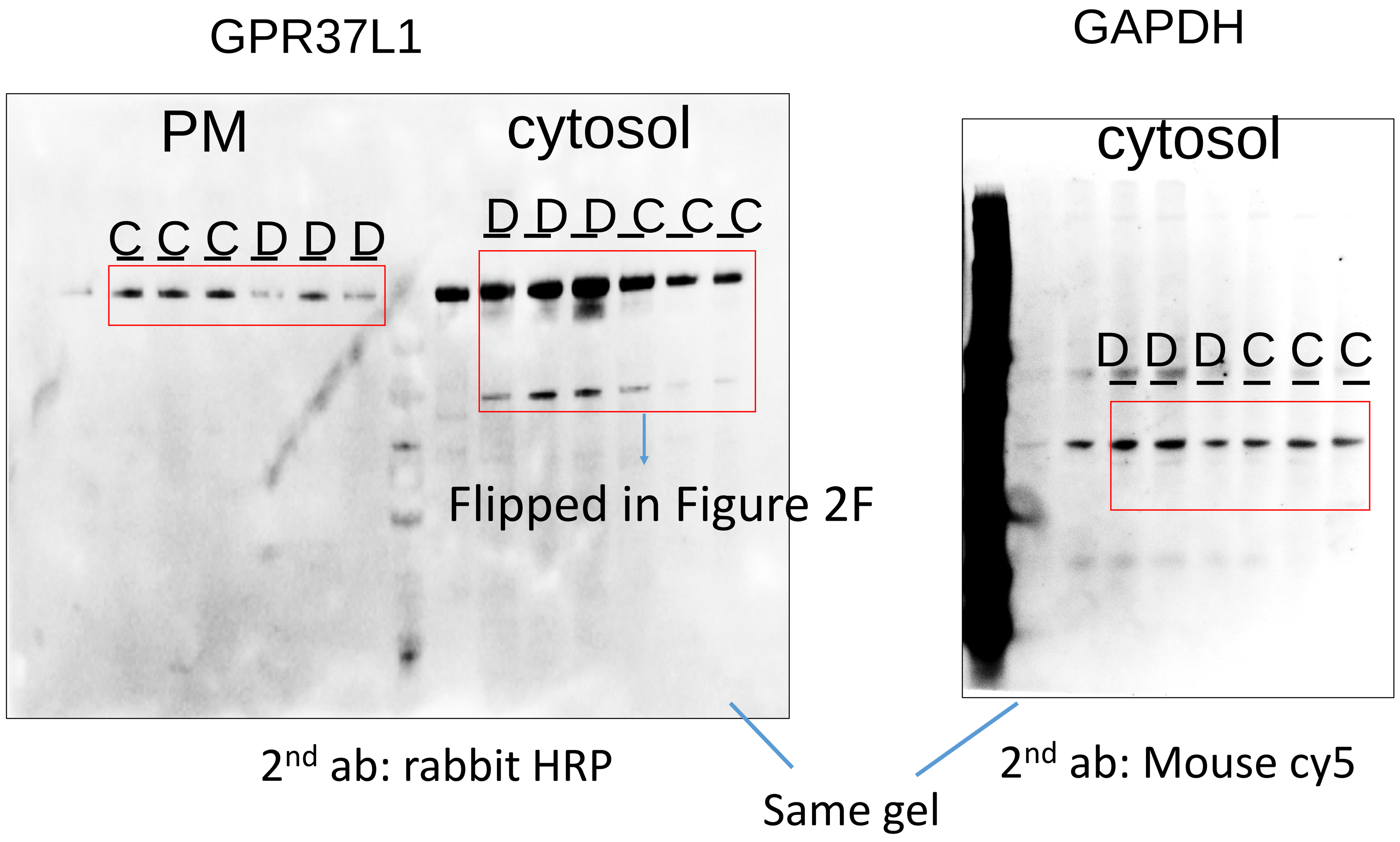

C: control
D: DPN

Figure 3C

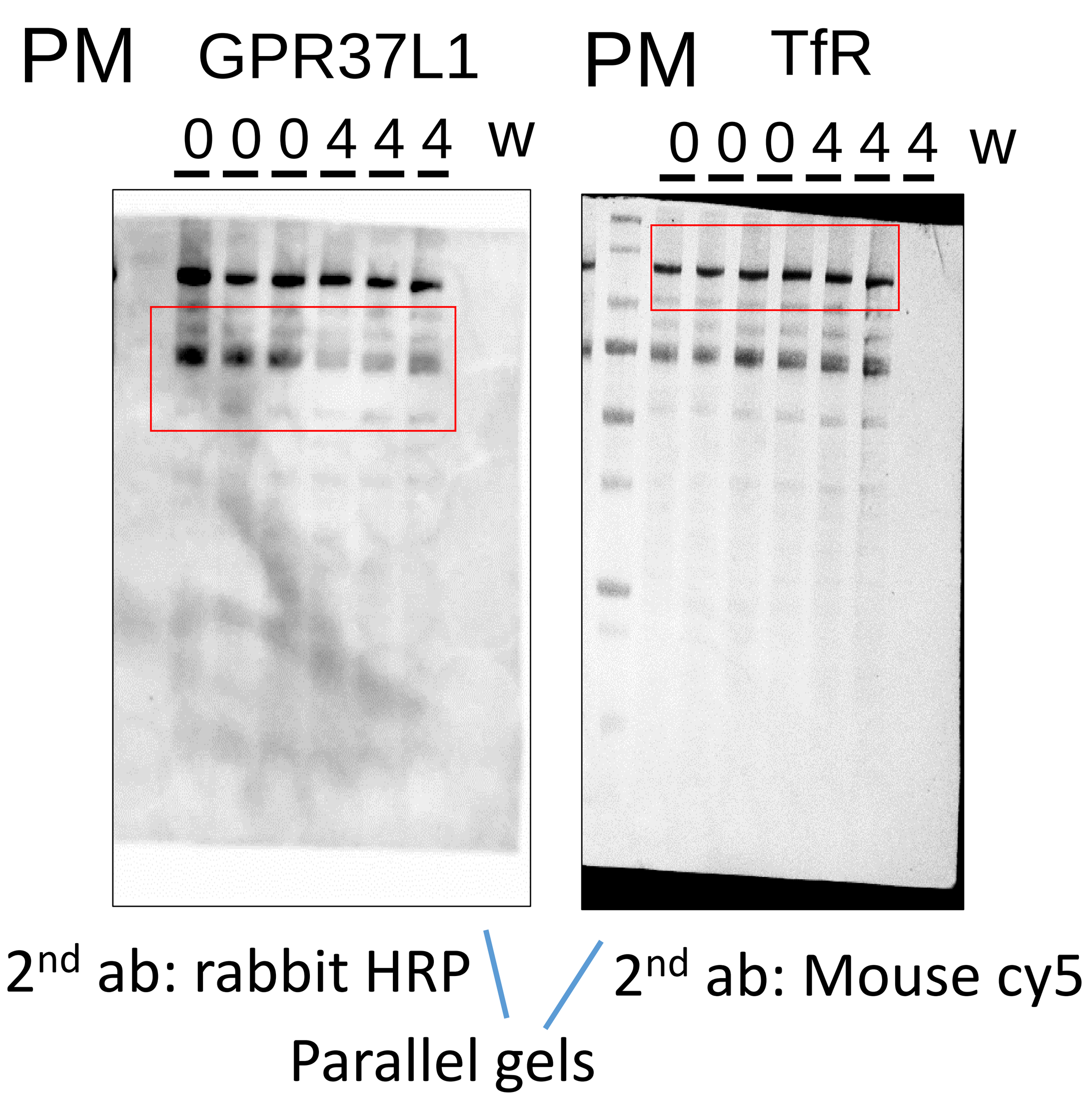

Figure 4C

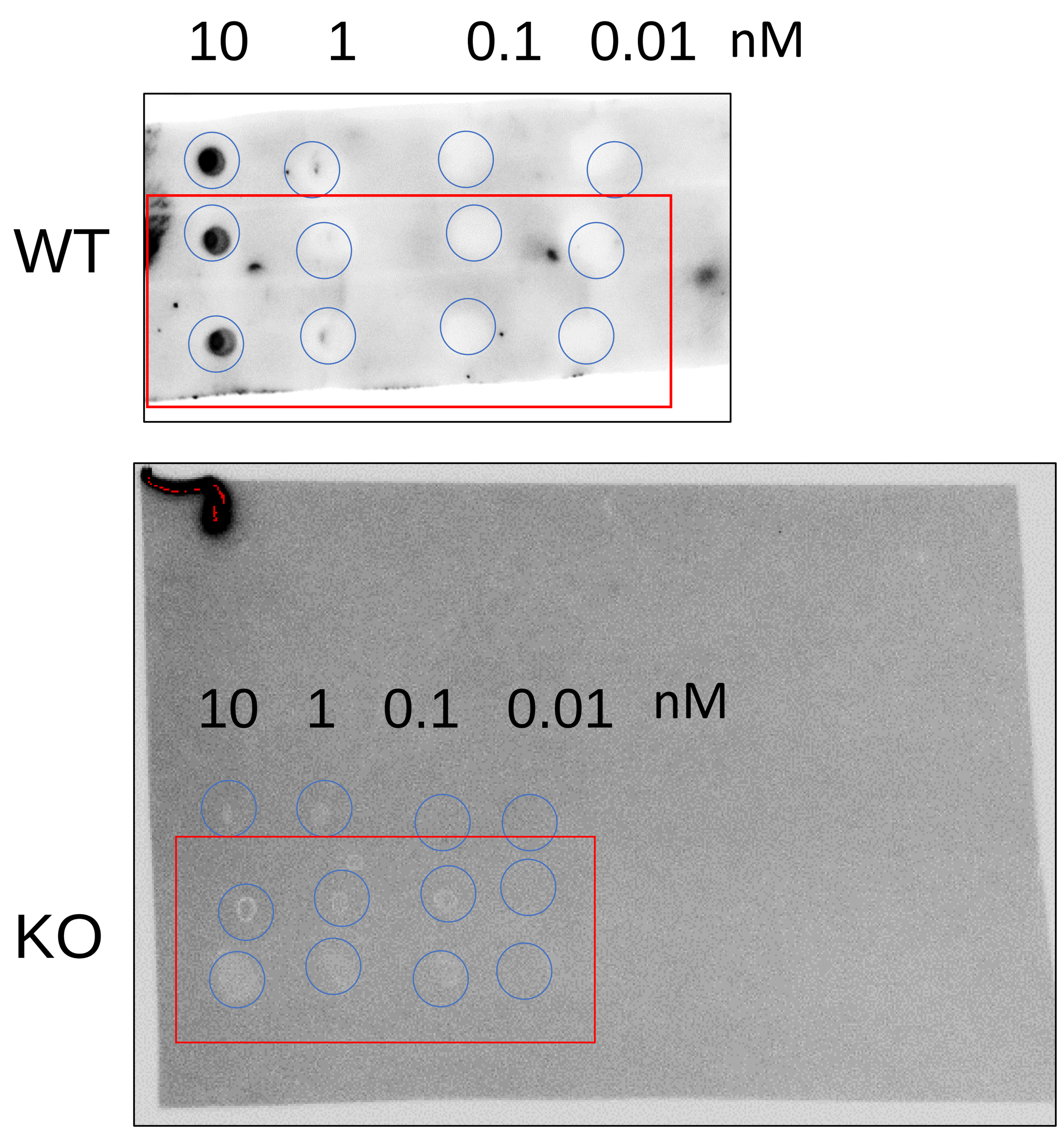

Figure 6B

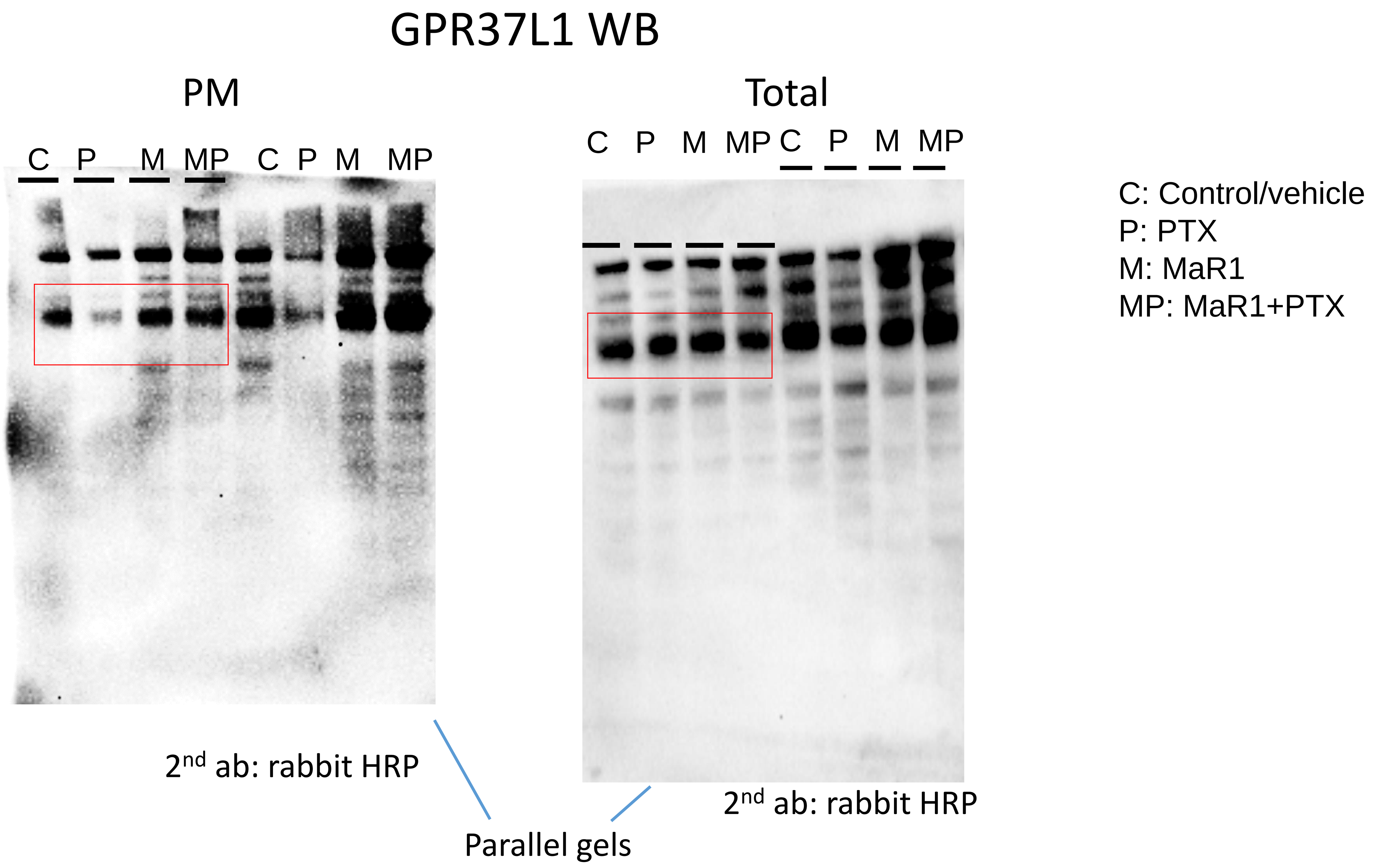

Two repeats are shown in the same gels.

Figure 6L

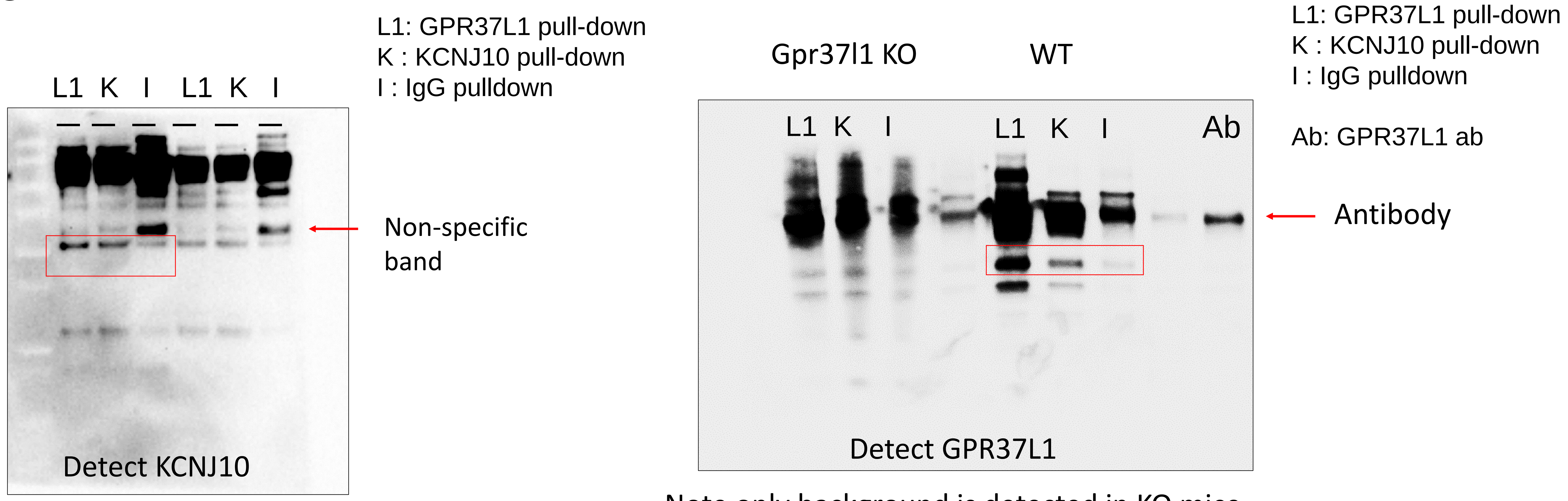

Two repeats are shown in the same gel.

Note only background is detected in KO mice.

Figure 7E

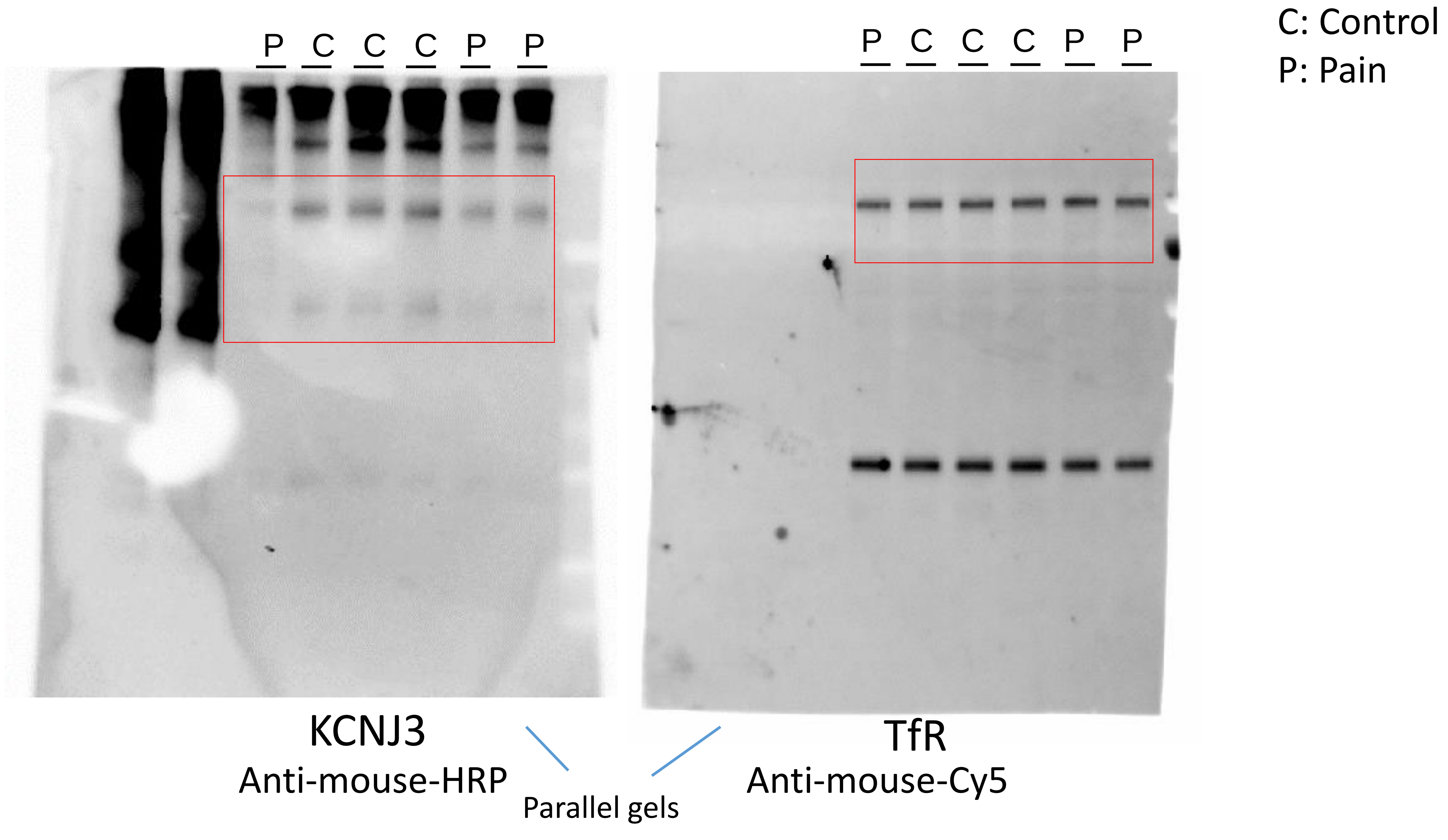

Fig. S4B

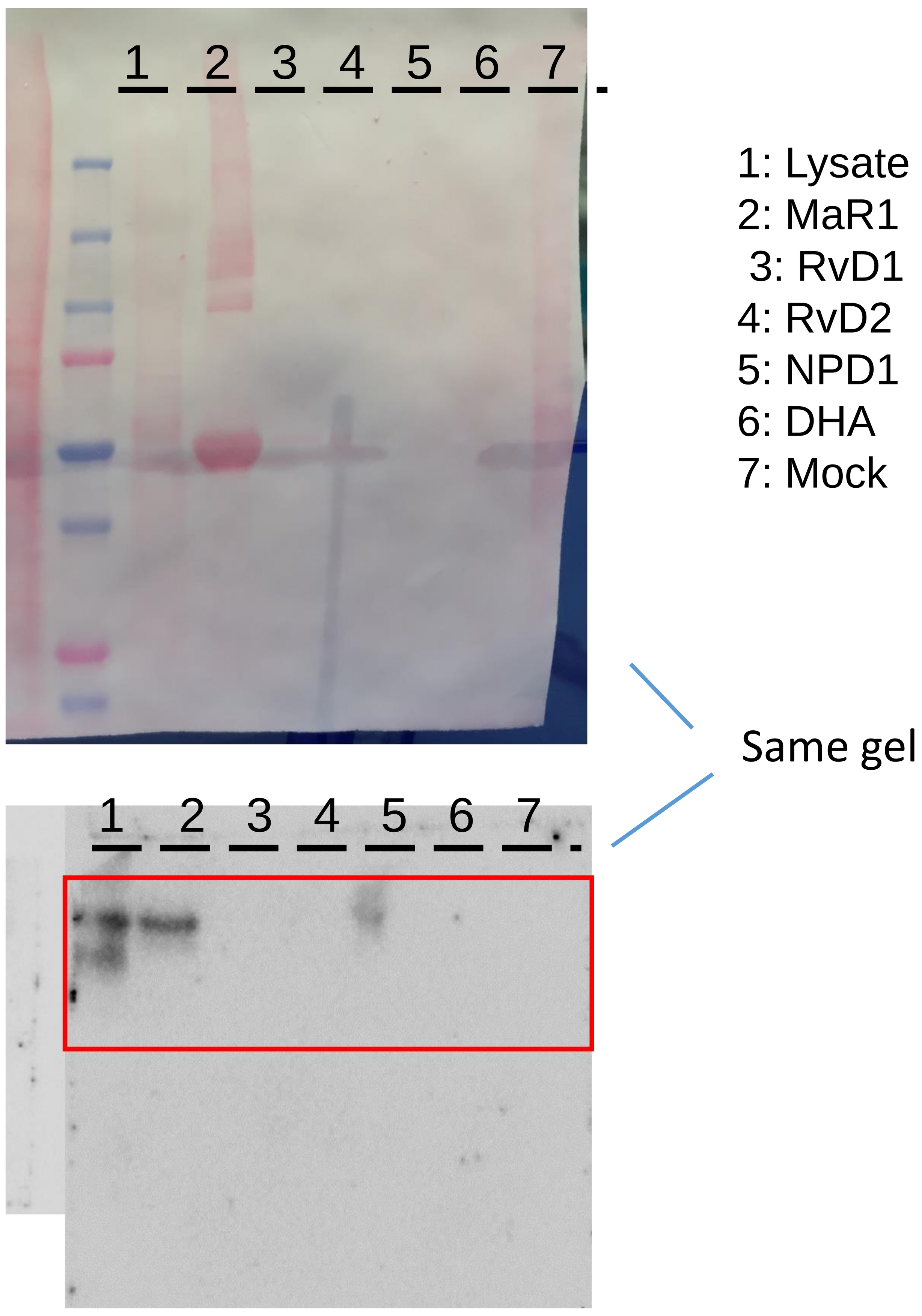

Fig. S6A

Time after PTX treatment in whole-mount DRG

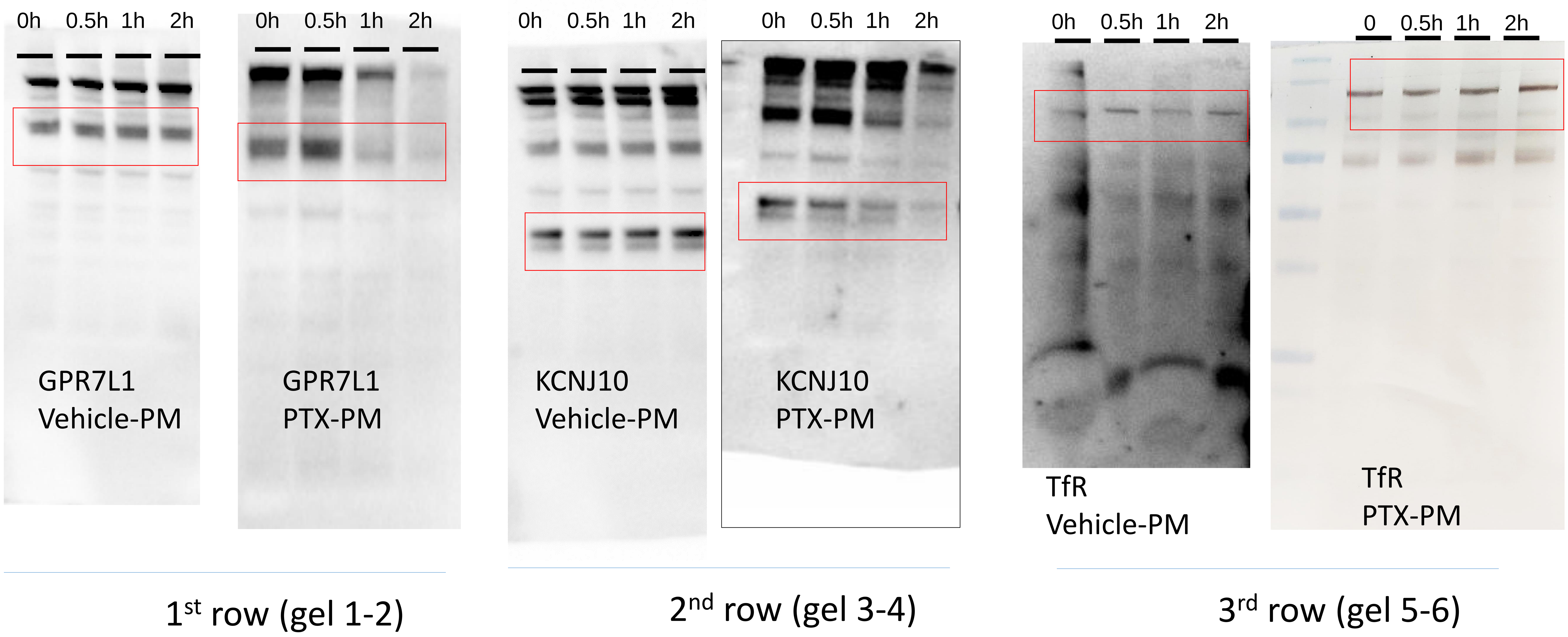

Gels 1,3,5 and gels 2,4,6 are parallel gels respectively.

Fig. S9C-D

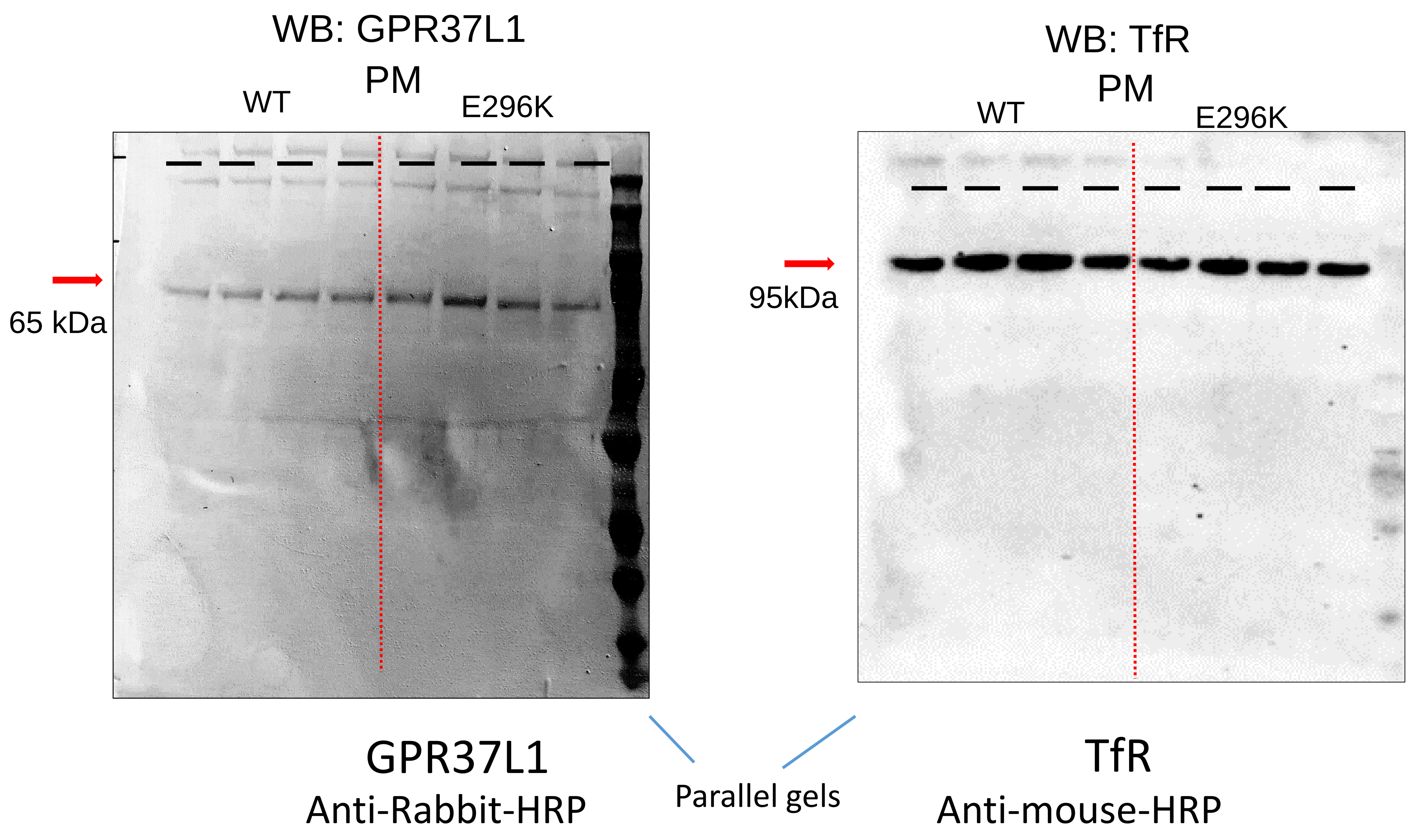

WB: GPR37L1
PM
WT
E296K
65 kDa
WB: TfR
PM
WT
E296K
95kDa
GPR37L1
Anti-Rabbit-HRP
Parallel gels
TfR
Anti-mouse-HRP
